# TATN-1 Mutations Reveal a Novel Role for Tyrosine as a Metabolic Signal That Influences Developmental Decisions and Longevity in *Caenorhabditis elegans*

**DOI:** 10.1371/journal.pgen.1004020

**Published:** 2013-12-19

**Authors:** Annabel A. Ferguson, Sudipa Roy, Kaitlyn N. Kormanik, Yongsoon Kim, Kathleen J. Dumas, Vladimir B. Ritov, Dietrich Matern, Patrick J. Hu, Alfred L. Fisher

**Affiliations:** 1 Division of Geriatric Medicine, Department of Medicine, University of Pittsburgh, Pittsburgh, Pennsylvania, United States of America; 2 Department of Medicine, University of Texas Health Science Center at San Antonio, San Antonio, Texas, United States of America; 3 Center for Healthy Aging, University of Texas Health Science Center at San Antonio, San Antonio, Texas, United States of America; 4 Life Sciences Institute, University of Michigan, Ann Arbor, Michigan, United States of America; 5 Department of Environmental and Occupational Health, Graduate School of Public Health, University of Pittsburgh, Pittsburgh, Pennsylvania, United States of America; 6 Biochemical Genetics Laboratory, Department of Laboratory Medicine and Pathology, Mayo Clinic College of Medicine, Rochester, Minnesota, United States of America; 7 Departments of Internal Medicine and Cell and Developmental Biology, University of Michigan Medical School, Ann Arbor, Michigan, United States of America; 8 GRECC, South Texas VA Health Care System, San Antonio, Texas, United States of America; Stanford University Medical Center, United States of America

## Abstract

Recent work has identified changes in the metabolism of the aromatic amino acid tyrosine as a risk factor for diabetes and a contributor to the development of liver cancer. While these findings could suggest a role for tyrosine as a direct regulator of the behavior of cells and tissues, evidence for this model is currently lacking. Through the use of RNAi and genetic mutants, we identify *tatn-1*, which is the worm ortholog of tyrosine aminotransferase and catalyzes the first step of the conserved tyrosine degradation pathway, as a novel regulator of the dauer decision and modulator of the *daf-2* insulin/IGF-1-like (IGFR) signaling pathway in *Caenorhabditis elegans*. Mutations affecting *tatn-1* elevate tyrosine levels in the animal, and enhance the effects of mutations in genes that lie within the *daf-2*/insulin signaling pathway or are otherwise upstream of *daf-16*/FOXO on both dauer formation and worm longevity. These effects are mediated by elevated tyrosine levels as supplemental dietary tyrosine mimics the phenotypes produced by a *tatn-1* mutation, and the effects still occur when the enzymes needed to convert tyrosine into catecholamine neurotransmitters are missing. The effects on dauer formation and lifespan require the *aak-2*/AMPK gene, and *tatn-1* mutations increase phospho-AAK-2 levels. In contrast, the *daf-16*/FOXO transcription factor is only partially required for the effects on dauer formation and not required for increased longevity. We also find that the controlled metabolism of tyrosine by *tatn-1* may function normally in dauer formation because the expression of the TATN-1 protein is regulated both by *daf-2*/IGFR signaling and also by the same dietary and environmental cues which influence dauer formation. Our findings point to a novel role for tyrosine as a developmental regulator and modulator of longevity, and support a model where elevated tyrosine levels play a causal role in the development of diabetes and cancer in people.

## Author Summary

In people, elevated blood levels of the amino acid tyrosine are seen in obese individuals, and these elevations represent a novel risk factor for the development of diabetes. The enzyme tyrosine aminotransferase, which removes tyrosine from the body, has also been identified as a tumor suppressor gene, and this enzyme normally acts to prevent the development of liver cancer. In our work, we identify tyrosine aminotransferase as a regulator of larval development and adult longevity in the non-parasitic worm *Caenorhabditis elegans*. Worms with mutations impairing tyrosine aminotransferase activity show elevated levels of tyrosine, are prone to arresting development in a larval stage called a dauer, and show increased longevity. Part of the effect of tyrosine aminotransferase is due to inhibitory effects on an insulin-like signaling pathway in the worms. Our work suggests that levels of the amino acid tyrosine are sensed and can lead to changes in cell signaling. These results may provide insights into how tyrosine could be involved in obesity, diabetes, and cancer in people.

## Introduction

The aromatic amino acid tyrosine serves many metabolic roles including being a building block for protein synthesis, a source of energy, and a precursor for the synthesis of melanin and several neurotransmitters including dopamine and other catecholamines. Beyond these currently known functions for tyrosine, recent work has suggested that tyrosine could also play regulatory roles in both metabolism and the control of cell proliferation. Specifically, in people elevated serum tyrosine levels occur with obesity and represent a risk factor for the development of diabetes [1]–[6]. Additionally, the enzyme tyrosine aminotransferase (TAT), which acts to normally convert tyrosine to energy, has been identified as a tumor suppressor gene which acts to promote apoptosis and prevent the development of hepatocellular carcinoma [7]. How changes in tyrosine metabolism could contribute to these disease processes is currently unknown, but it is possible that levels of this amino acid could play a direct regulatory role for the behavior of specific cells and tissues. While consistent with the available data, direct evidence for this model is currently lacking.

The nematode *Caenorhabditis elegans* normally progresses through four larval stages before developing into a reproductive adult animal. However specific cues, such as crowding, low food availability, or elevated temperature, can be sensed by the developing worm and lead to developmental arrest in a diapause state called a dauer larva [8]–[11]. Entry into dauer permits worms to delay the completion of development and the initiation of reproduction in environments which are not favorable, and instead the animals can survive as a dauer for up to several months before resuming normal development when conditions become favorable for reproductive success. This developmental decision requires a complicated interplay of sensory neurons with specific cGMP, TGF-β, and insulin-like signaling cascades controlling the choice of reproductive versus dauer development [9], [10].

In the worm, the *daf-2* insulin/IGF-1 receptor (IGFR) signaling pathway is involved in both dauer development and adult longevity [12]–[14]. Active signaling through the pathway during development enables animals to reach reproductive adulthood whereas reductions in *daf-2/*IGFR signaling due to either environmental triggers or genetic mutations lead to arrest as a dauer [9], [14]. In adult worms, *daf-2/*IGFR signaling is a major modulator of longevity and mutations impairing the pathway can result in 100% increases in lifespan [13].

**Figure 1:**
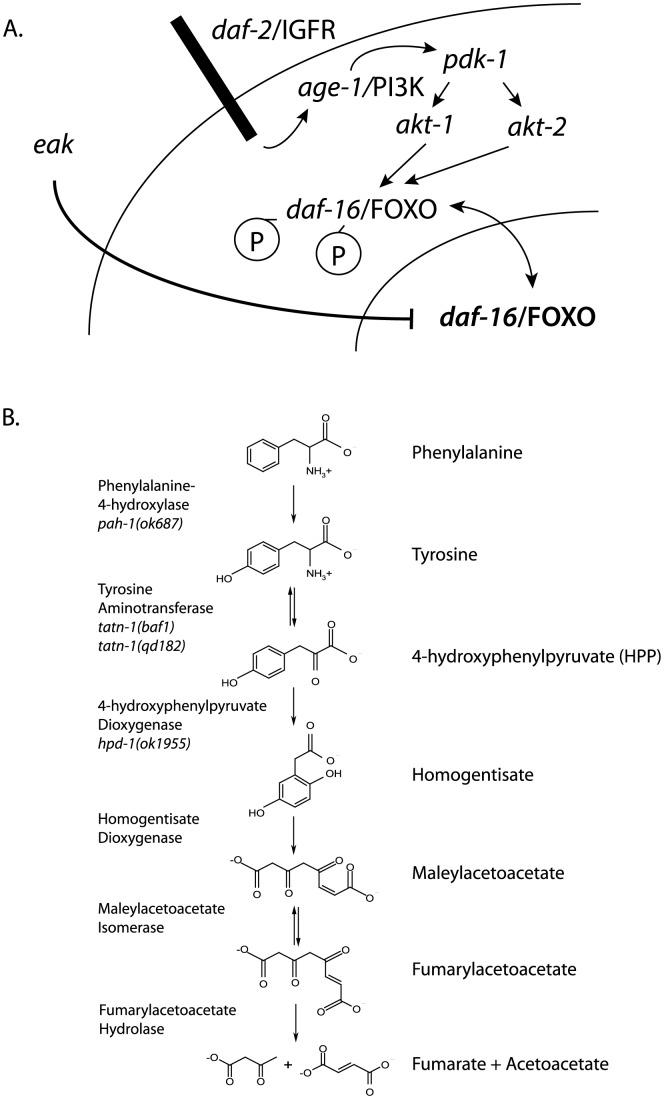
Diagram of *daf-2*/IGFR signaling pathway and tyrosine metabolic pathway. (A) For dauer formation in *C. elegans* the *daf-2*/IGFR receptor lies upstream of a PI3 kinase signaling cascade consisting of *age-1*/PI3 kinase, the phosphoinoside-dependent kinase *pdk-1*, and the protein kinase B family kinases *akt-1* and *akt-2*. Both AKT-1 and AKT-2 act to phosphorylate DAF-16/FOXO and prevent entry of this protein into the nucleus. Inhibition of AKT-1 leads to entry of DAF-16/FOXO into the nucleus without the activation of DAF-16 target genes. This suggested that other pathways acted to control the transcriptional activity of nuclear DAF-16/FOXO. The *eak* genes are a group of structurally unrelated genes which act in a cell non-autonomous manner to restrain the transcriptional activity of nuclear DAF-16/FOXO through an undefined molecular mechanism. (B) Tyrosine is degraded to fumarate and acetoacetate via a five step degradation pathway. Shown are the names and structures of the intermediates as well as the names of the enzymes which catalyze each step. Also shown are mutant alleles affecting enzymes in the pathway that are studied in this work.

At a molecular level, the *daf-2/*IGFR pathway consists of *daf-28* and other insulin-like peptides, which are thought to act as ligands for the DAF-2 insulin/IGF-1 receptor [15]–[18]. Downstream of *daf-2/*IGFR, is the *age-1* PI3 kinase and a kinase cascade consisting of the phosphoinositide-dependent kinase *pdk-1* and the protein kinase B genes *akt-1* and *akt-2* (Figure 1A) [19]–[22]. Both *akt-1* and *akt-2* normally act to phosphorylate the DAF-16 FOXO transcription factor which leads to its retention in the cytoplasm [23]–[25]. Reductions in either *daf-2*/IGFR or combined *akt-1* and *akt-2* activity result in the entry of DAF-16/FOXO into the nucleus and strong activation of DAF-16/FOXO target genes [23]–[25]. In contrast, loss of only *akt-1* activity leads to the translocation of DAF-16/FOXO into the nucleus but a lesser increase in the expression of DAF-16/FOXO target genes (Figure 1A and Table S1) [26], [27]. This finding suggested that additional pathways could be involved in controlling the transcriptional activity of DAF-16/FOXO (Figure 1A). One group of potential regulators is the *eak* (enhancer of *akt-1* null) genes, which were identified in a forward genetic screen and act in a non-cell autonomous manner to control the transcriptional activity of nuclear localized *daf-16*/FOXO (Figure 1A) [26]–[29]. The identified *eak* genes lack structural homology to one another and appear to lie in one or more poorly characterized pathways that act in parallel to *akt-1*. The identification of the *eak* genes suggests that additional novel pathways either downstream or parallel to insulin signaling may await discovery.

In both vertebrates and worms, there is data suggesting a link between tyrosine metabolism and insulin signaling. TAT, which catalyzes the first step in a conserved degradation pathway that converts tyrosine to fumarate and acetoacetate (Figure 1B), has been well studied as a target of regulation by insulin signaling in vertebrates with insulin effects seen at both the transcriptional and translational level [30]–[38]. Further, in *C. elegans* the *hpd-1* gene, which encodes the enzyme 4-hydroxyphenylpyruvate dioxygenase and lies immediately downstream of TAT in the tyrosine degradation pathway, is a target gene for the *daf-16*/FOXO transcription factor and positively regulated by *daf-2*/IGFR signaling [39]. The down-regulation of *hpd-1* in *daf*-2/IGFR mutants could lead to a reduction in tyrosine clearance and could, at least in part, account for the increases tyrosine levels observed in these animals [40]. Furthermore, the inhibition of *hpd-1* by RNAi was shown to both extend lifespan and delay dauer exit through unknown mechanisms [39]. Hence, tyrosine metabolism appears to be actively controlled by insulin signaling though the consequences of this regulation are currently unclear.

In our work, we identify *tatn-1*, which is the worm ortholog of TAT, to be a novel dauer formation regulator that is under the control of several dauer-inducing stimuli, including *daf-2*/IGFR signaling, and ultimately regulates tyrosine levels in the worm. We further find that *tatn-1* mutations enhance the dauer-formation and lifespan phenotypes of both *daf-2*/IGFR and *eak* mutants suggesting that elevated tyrosine levels have inhibitory effects on insulin signaling. These effects require the *aak-2*/AMPK gene, and *tatn-1* mutants have elevated levels of the activated phospho-AAK-2 protein consistent with activation of AAK-2 signaling in response to elevated tyrosine. The activation of AAK-2 may lead to effects on the downstream transcription factors *daf-16*/FOXO and *crh-1*/CREB. We see a partial dependence on *daf-16*/FOXO for some *tatn-1* phenotypes and activation of *daf-16*/FOXO target genes, and the loss of *crh-1*/CREB, which is inhibited by activated *aak-2*, mimics some *tatn-1* phenotypes. Together our findings establish a novel role for tyrosine as a metabolic signal that influences insulin signaling, development, and lifespan through effects on *aak-2*/AMPK signaling. While further study is necessary, our results also suggest that the recently observed associations between tyrosine metabolism and both diabetes and cancer are due to elevated tyrosine levels playing a direct causal role in disease pathogenesis.

## Results

### Reduced tyrosine aminotransferase activity promotes dauer arrest

**Figure 2:**
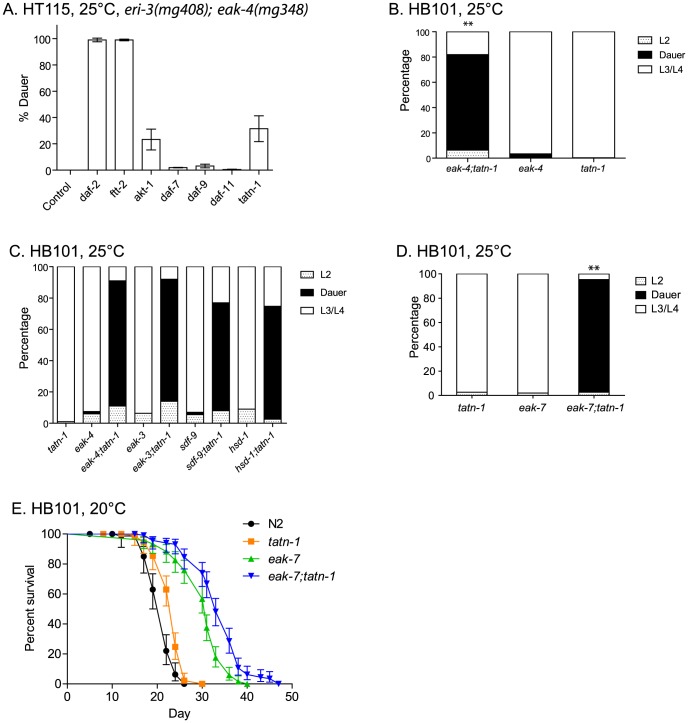
Tyrosine aminotransferase mutations enhance *eak* dauer and lifespan phenotypes. (A) Effect of treatment of *eri-3(mg408); eak-4(mg348)* mutants with RNAi clones for *daf-2*, *ftt-2*, *akt-1*, *daf-7*, *daf-9*, *daf-11*, and *tatn-1* on dauer arrest at 25°C. Bars represent mean percentage of dauers observed in two trials, and the error bars represent standard deviation. The empty vector RNAi vector was used as a negative control. (B) Enhanced dauer formation by *eak-4(mg348);tatn-1(baf1)* versus the *eak-4(mg348)* or *tatn-1(baf1)* mutations alone. ** p<0.001 for pairwise Fisher's exact test. (C) *tatn-1* mutations enhance dauer arrest by *eak-3(mg344)*, *sdf-9(mg337)*, and *hsd-1(mg337)* mutants. ** p<0.001 for each double mutant compared to its respective *eak* single mutant by Fisher's exact test. (D) *tatn-1* enhances *eak-7(tm3188)* dauer arrest. ** p<0.001 for comparisons between *eak-7;tatn-1* and either *tatn-1* or *eak-7* alone. (E) *tatn-1(baf1)* extends the lifespan of both wild type and *eak-7* worms. Shown are survival curves for wild type N2, *tatn-1(baf1)*, *eak-7(3188)*, and *eak-7(3188); tatn-1(baf1)*, with error bars showing the 95% confidence intervals for each point. The mean survival for N2, *tatn-1*, *eak-7*, and *eak-7;tatn-1* are 21.0, 23.0, 30.0, and 33.5 days, respectively. p<0.001 for all comparisons by log-rank test.

The *eak* genes were identified in a genetic screen as enhancers of the weak dauer formation phenotype shown by *akt-1* mutants, and these genes normally act to suppress the transcriptional activity of nuclear localized *daf-16*/FOXO [26]–[29]. To identify new genes that act in parallel to *eak* genes to control dauer formation, we performed a genome-wide RNAi screen for gene inactivations that enhance the weak dauer-constitutive phenotype of the *eak-4(mg348)* mutant. Since RNAi of dauer-constitutive genes typically yields a weaker phenotype than the corresponding mutants, we constructed an *eri-3(mg408); eak-4(mg348)* double mutant to enhance the sensitivity of the *eak-4* mutant to RNAi [41], [42]. Control experiments demonstrated that RNAi inhibition of *daf-2*/IGFR, *akt-1*, or the 14-3-3 gene *ftt-2* enhance dauer arrest by the *eri-3; eak-4* mutants whereas RNAi inhibition of *daf-7*, *daf-9*, or *daf-11*, which encode components of TGF-β, dafachronic acid, and cGMP pathways, respectively, do not (Figure 2A). These data suggested that the screen could be enriched for genes that act in the *daf-2*/IGFR pathway to control dauer arrest.

Among the RNAi clones identified from the screen was *tatn-1*, which encodes the worm ortholog of tyrosine aminotransferase. Inhibition of *tatn-1* by RNAi enhanced dauer arrest by *eri-3; eak-4* mutants to the same extent as *akt-1* RNAi (Figure 2A) [43]. Tyrosine aminotransferase is the first enzyme in the conserved five step tyrosine degradation pathway present in worms and other eukaryotes, and this enzyme catalyzes the deamination of tyrosine to produce 4-hydroxyphenylpyruvate (Figure 1B). The subsequent steps in the degradation pathway convert 4-hydroxyphenylpyruvate into fumarate and acetoacetate which can be ultimately metabolized by the Krebs' cycle (Figure 1B).

Similarly to the effects of *tatn-1* RNAi, we also found that the *tatn-1(baf1)* mutation, which has a P224S mutation in a conserved region of the protein and is likely a partial loss of function allele [43], [44], also promoted dauer arrest by the *eak-4(mg348)* mutants (Figure 2B and Table S2). Interestingly, the interaction between *tatn-1* and *eak-4* was strongly influenced by worm diet with diets consisting of the *E. coli* K12-derived HT115 or K12-B hybrid HB101 bacterial strains showing the strongest interaction (Figure S1). Additionally, we observed that a second *tatn-1* allele, *tatn-1(qd182)*, both enhanced dauer arrest by the *eak-4* mutant and also had a weak effect on dauer formation in isolation (Figure S2). The *tatn-1(qd182)* allele encodes a protein with a G171E mutation affecting a highly conserved glycine residue (Figure S2). Together these findings demonstrate a novel role for tyrosine aminotransferase as a regulator of the dauer development decision.

We then tested whether *tatn-1* interacted with other *eak* genes via the construction of *tatn-1; eak* mutants. We found that *tatn-1(baf1)* enhanced the dauer-constitutive phenotype of all *eak* mutants tested, including *eak-4(mg348), eak-3(mg344),eak-5/sdf-9(mg337), eak-2/hsd-1(mg433)*, and *eak-7(tm3188)* (Figure 2C and 2D). *eak-7* showed a particularly strong interaction with *tatn-1*, with 92.4% of the population forming dauers at 25°C (Figure 2D), and we observed dauers in *eak-7; tatn-1* in cultures growing at lower temperatures or on other worm diets. These data suggest that *tatn-1* is a general enhancer of dauer formation by *eak* mutants.

In addition to enhancing dauer formation, *eak-7* mutations, but not other *eak* mutations, extend the lifespan of wild-type and *akt-1* mutant worms [27]. We tested whether a *tatn-1* mutation also enhanced the lifespan of *eak-7* mutants by conducting survival assays using wild type N2, *tatn-1(baf1), eak-7(tm3188), and eak-7(tm3188); tatn-1(baf1)* worm populations. We found that *tatn-1*, *eak-7*, and *eak-7; tatn-1* mutants all showed increased longevity relative to N2 (mean survivals of 21.0, 23.0, 30.0, and 33.5 days, respectively) (Figure 2E and Table S3). Specifically, *tatn-1* extends mean survival 10.4%, *eak-7* extends mean survival 43.1%, and *eak-7; tatn-1* increases survival 59.2% over wild type (Figure 2E). These findings demonstrate a novel role for *tatn-1* in modulating lifespan and also demonstrate that the effects of the *eak-7* – *tatn-1* genetic interaction also influence adult longevity.

### Tyrosine aminotransferase interacts with insulin signaling in worms

**Figure 3:**
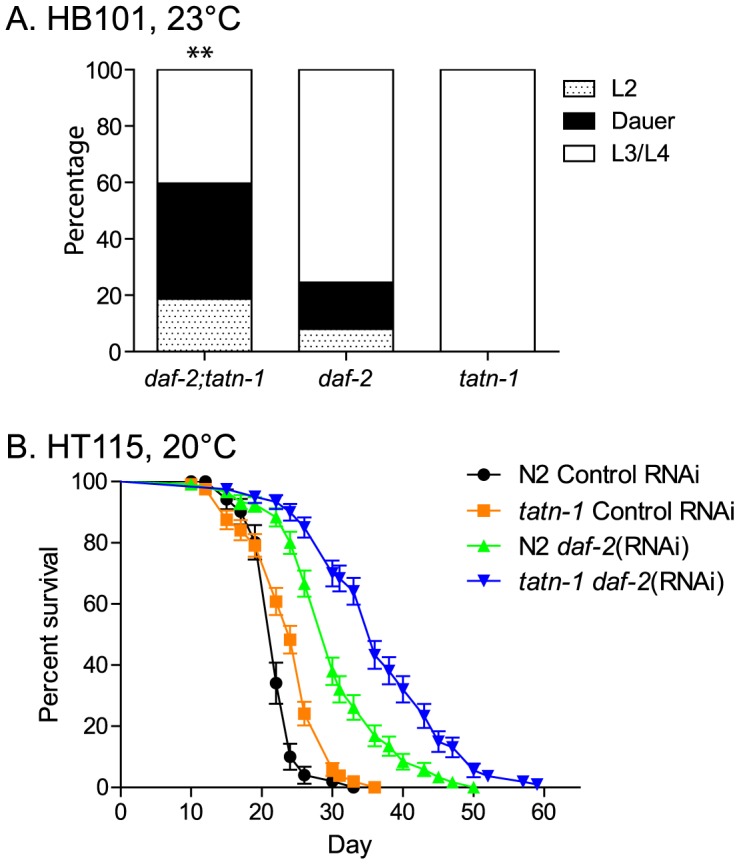
The *tatn-1* mutant enhances dauer formation and lifespan of worms with impaired *daf-2*/IGFR signaling. (A) Enhanced dauer formation by *daf-2(e1368); tatn-1(baf1)* compared to *daf-2(e1368)* grown at 23°C for 3 days. ** p<0.01 by Fisher's exact test. (B) *tatn-1* extends the lifespan of worms treated with *daf-2* RNAi from day 1 of adulthood. The mean survival for N2 control RNAi, *tatn-1(baf1)* control RNAi, N2 *daf-2(RNAi)*, and *tatn-1(baf1) daf-2(RNAi)* are 22.0, 24.0, 30.5, and 37.0 days, respectively. p<0.001 for N2 vs. *tatn-1* on control RNAi and N2 *daf-2(RNAi)* vs. *tatn-1 daf-2(RNAi)* by log-rank test.

Mutations in the *eak* genes enhance the dauer formation phenotype of loss-of-function mutations affecting genes in the *daf-2*/IGFR signaling pathway, so we tested whether *tatn-1* mutations also enhanced *daf-2*/IGFR mutant phenotypes [28]. We used the *daf*-2*(e1368)* allele which has a strong dauer-constitutive phenotype when grown at 25°C but a weaker phenotype when grown at lower temperatures. Compared to *tatn-1(baf1)* and *daf-2(e1368)* alone, we found that *daf-2(e1368); tatn-1(baf1)* mutants showed increased levels of dauer formation when grown at 23°C (Figure 3A). Further, we found that the *tatn-1* mutation extends the adult lifespan of worms treated with *daf-2* RNAi starting at day 1 of adulthood (Figure 3B). We used RNAi treatment due to the high levels of dauer arrest that we observed with the *daf-2(e1368); tatn-1(baf1)* animals. In these RNAi experiments, the mean survival of worms were 22.0 days for N2 on control RNAi, 24.0 days for *tatn-1* on control RNAi, 30.5 days for N2 on *daf-2* RNAi, and 37.0 days for *tatn-1* on *daf-2* RNAi (Figure 3B and Table S3). Hence, while *daf-2* RNAi treatment extends the survival of wild type N2 worms by 37.4%, the inclusion of the *tatn-1* allele further extends the lifespan of *daf-2*(RNAi) treated worms by an additional 21%. These findings show a genetic interaction between *tatn-1* and *daf-2*/IGFR with impaired tyrosine degradation enhancing the *daf-2*/IGFR dauer formation and lifespan phenotypes.

### *tatn-1* likely does not alter PI3 kinase or TGF-β signaling

**Figure 4:**
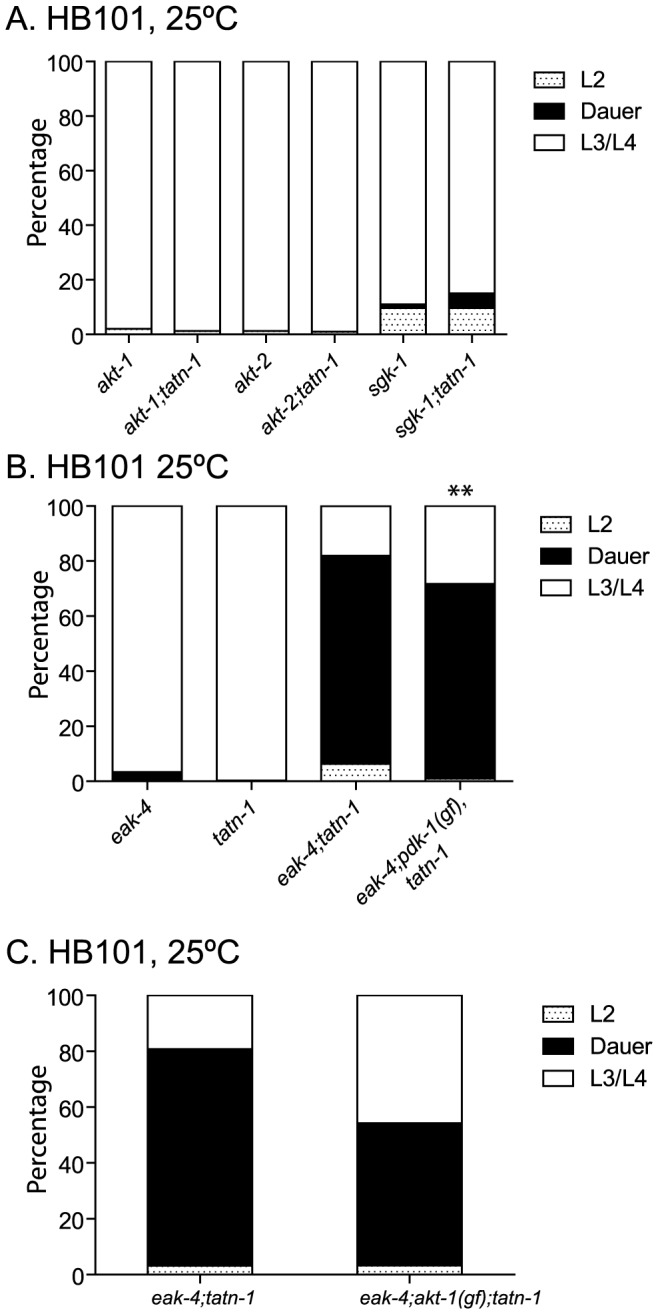
*tatn-1* effects on development do not require changes in PI3 kinase signaling. (A) *tatn-1(baf1)* does not augment dauer arrest by mutants with loss-of-function mutations in the protein kinase B genes *akt-1*, *akt-2*, or *sgk-1*. (B) The *pdk-1(mg142)* gain-of-function allele minimally affects dauer formation by *eak-4(mg348); tatn-1(baf1)* mutants. % dauers observed was 75.6% in *eak-4; tatn-1* and 70.7% in *eak-4; pdk-1(mg142); tatn-1*. ** p<0.001 by Fisher's exact test. (C) The *akt-1(mg144)* gain-of-function allele weakly inhibited dauer formation by *eak-4(mg348); tatn-1(baf1)* mutants. % dauers observed was 77.6% in *eak-4; tatn-1* and 51% in *eak-4; akt-1(mg144); tatn-1*.

Since the *eak* mutations interact with *akt-1* mutations to enhance dauer formation, the effects of *tatn-1* mutations could be due to inhibition of the PI3 kinase signaling pathway (Figure 1A) [20]–[22], [28], [45]. To test this possibility, we looked for genetic interactions between loss-of-function and gain-of-function mutations affecting the PI3 kinase pathway with *tatn-1*. First, we constructed *tatn-1* mutants containing the loss-of-function mutations affecting the PKB genes *akt-1* and *akt-2*, and the related kinase *sgk-1*. We found that none of these genes interacts strongly with a *tatn-1* mutation to enhance dauer arrest (Figure 4A). This finding suggests that *tatn-1* is not an upstream regulator of either *akt-1* or *akt-2*. To further test whether tyrosine affected the PI3 kinase signaling cascade, we tested whether the *eak-4(mg348); tatn-1(baf1)* interaction was blocked by gain-of-function mutations in either *pdk-1* or *akt-1*. These mutations were identified in genetic screens as suppressors of the dauer-constitutive phenotype of an *age-1*/PI3K null mutant [20], [21]. We constructed both *eak-4(mg348); pdk-1(mg142), tatn-1(baf1)* and *eak-4(mg348); akt-1(mg144); tatn-1(baf1)* mutants and examined the effects of these mutations on dauer formation. We found that *pdk-1(mg142)*, which is a dominant gain-of-function allele of 3-phosphoinositide-dependent kinase and lies upstream of *akt-1*, *akt-2*, and *sgk-1*, had little effect on dauer formation by the *eak-4(mg348); tatn-1(baf1)* mutants (70% with *pdk-1(mg142)* versus 75% without) (Figure 4B). We further found that *akt-1(mg144)* had at most a modest effect on dauer formation by *eak-4(mg348); tatn-1(baf1)* mutants (51% with *akt-1(mg144)* versus 77.6% without) (Figure 4C). Together these results suggest that the interaction between *eak-4* and *tatn-1* likely does not involve changes in the PI3 kinase signaling cascade.

In addition to the *daf-2*/IGFR signaling pathway, dauer formation in worms is also regulated by a TGF-β signaling pathway [9]–[11]. While traditionally these two pathways are viewed as independent, more recent work has indicated that cross-talk between the pathways may occur. Specifically, genes in one pathway, such as *sdf-9/eak-5* or *pdp-1*, have been shown to augment the dauer formation phenotypes of genes in the other pathway [46], [47]. Notably, the *pdp-1* phosphatase was identified from an RNAi screen using *daf-2*/IGFR pathway mutants as was *tatn-1*. To test whether *tatn-1* could act in the TGF-β signaling pathway, we blocked this pathway with either the *daf-3/*SMAD or *daf-5*/Sno mutations [41], [48], [49]. We found that neither mutant reduced dauer formation by the *eak-4; tatn-1* mutants (Figure S3), which is consistent with *tatn-1* acting independently of the TGF-β pathway.

### AAK-2/AMPK is activated in *tatn-1* mutants and required for effects on dauer formation and longevity

**Figure 5:**
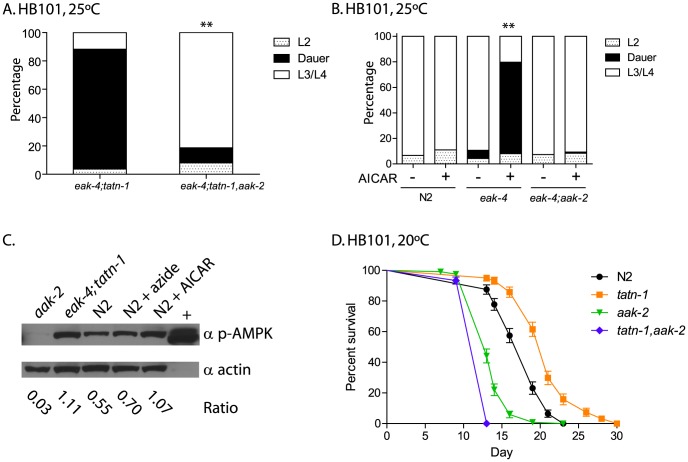
*aak-2* activity is necessary and sufficient for *tatn-1* effects on development and longevity. (A) The effects of *tatn-1(baf1)* on dauer formation require the *aak-2*/AMPK gene. ** p<0.001 by Fisher's exact test. (B) Activation of *aak-2* with the AMPK agonist AICAR (0.125 mM) mimics the effects of *tatn-1* on *eak-4(mg348)* mutants. The effect of AICAR depends on *aak-2*/AMPK because an *eak-4*; *aak-2* mutant fails to respond to AICAR. ** p<0.001 for comparison of *eak-4* with and without AICAR by Fisher's exact test. (C) Levels of phospho-AAK-2 are increased in *eak-4(mg348); tatn-1(baf1)* L2 larval worms or N2 L2 larval worms treated with 1 mM AICAR compared to untreated N2 L2 larval worms. Levels of phospho-AAK-2 was detected using an anti-phospho-T172 specific antibody, and actin was a loading control. The ratio represents the level of phospho-AMPK normalized for the actin level in each lane. N2 larval worms treated with 10 mM sodium azide to deplete ATP levels and the AMPK positive control extract (+) (Cell Signaling Technologies) were included as positive controls. (D) *aak-2* is required for *tatn-1* effects on lifespan. Mean survival for N2, *tatn-1(baf1)*, *aak-2(gt33)*, and *tatn-1(baf1); aak-2(gt33)* were 18.0, 21.0, 14.0, and 13.0 days, respectively. p<0.001 for all pairwise curve comparisons by log-rank test.

Since *tatn-1* did not interact with *akt-1, akt-2, or sgk-1*, we looked for alternate signaling pathways that could be activated by a *tatn-1* mutation, and then interact with the *daf-2*/IGFR signaling pathway. The AMP-activated protein kinase (AMPK) ortholog AAK-2 was considered as a candidate because *aak-2* interacts with *daf-2* signaling, plays roles in dauer development, modulates worm longevity, and acts in part through the *daf-16*/FOXO transcription factor which is part of the *daf-2*/IGFR signaling pathway [50]–[53]. To test the involvement of *aak-2*/AMPK, we compared dauer formation between *eak-4(mg348); tatn-1(baf1)* and *eak-4(mg348); tatn-1(baf1), aak-2(gt33)* mutants. We found that loss of *aak-2* strongly reduced dauer arrest from 84.7% to 10.7% (Figure 5A and Table S2). The *aak-2* mutation also reduced dauer formation by an *eak-4(mg348)*; *tatn-1(qd182)* mutant though to a lesser degree than with the *tatn-1(baf1)* allele (Figure S4). This may be due to the *tatn-1(qd182)* allele being a stronger loss-of-function allele than *tatn-1(baf1)* as suggested by the developmental delay phenotype shown by *tatn-1(qd182)* and the higher tyrosine levels found in this mutant (Figure S4 and below). Together these findings support a necessary role for *aak-2*/AMPK for worms to respond to reductions in *tatn-1* activity, especially with weaker alleles.

To further explore the role of *aak-2*/AMPK in dauer formation, we treated worms with the AMPK agonist AICAR. Treatment of wild-type N2 worms with 0.125 mM AICAR had no effect on development or dauer formation, but *eak-4* mutants treated with AICAR showed a significant increase in dauer formation compared to an untreated control (Figure 5B). This effect required *aak-2*/AMPK as an *eak-4(mg348); aak-2(gt33)* mutant failed to respond to AICAR (Figure 5B). These findings demonstrate that *aak-2* activity is both necessary and sufficient to promote dauer formation by *eak-4* mutants.

Our findings could suggest that AAK-2 is activated in the *tatn-1* mutants. To test this possibility we used western blotting to measure levels of AAK-2 phosphorylated on the activating Thr^243^ residue, which is analogous to Thr^172^ in the vertebrate orthologs. We found that treatment of N2 wild-type animals with either sodium azide which inhibits mitochondrial function and depletes ATP levels by approximately 50% at this dose or with 1 mM AICAR leads to increases in phosphorylated AAK-2 levels compared with untreated L2 larval N2 worms grown in parallel (Figure 5C) [54]. Furthermore levels of phospho-AAK-2 were also increased in *eak-4; tatn1* mutant L2 larvae compared to the N2 control larvae (Figure 5C). Importantly, the phopho-AAK-2 signal was lost in *aak-2(gt33)* mutants confirming the identity of this band as AAK-2 (Figure 5C). These findings demonstrate that both *tatn-1* mutations and AICAR treatment serve to activate AMPK in *C. elegans*, and that this activation of AAK-2 is required for the effects of *tatn-1* mutations on development.

The requirement for *aak-2*/AMPK for the effects of *tatn-1* could reflect impaired tyrosine degradation leading to reduced production of the TCA cycle precursors fumarate and acetoacetate and a consequent decrease in energy production. To test this possibility we analyzed worm lysates for levels of AMP and ATP. We found that *eak-4(mg348); tatn-1(baf1)* mutants had a lower AMP/ATP ratio than N2 worms grown in parallel (Figure S5). This suggests that the role of *aak-2*/AMPK does not reflect reduced energy production in the *tatn-1* mutants.

Finally, we tested whether the effect of *tatn-1(baf1)* on adult lifespan requires *aak-2* activity, and we found that a *tatn-1* mutation increased wild-type lifespan by 17.8%, but decreased *aak-2(gt33)* lifespan by 8.9% compared to *aak-2(gt33)* alone (N2 mean survival 18.0 days, *tatn-1(baf1)* 21.0 days, *aak-2(gt33)* 14.0 days, and *tatn-1(baf1), aak-2(gt33)* 13.0 days) (Figure 5C and Table S3). Hence *aak-2* is both required for the *tatn-1* lifespan increase and may even play a protective role in animals with reduced *tatn-1* activity.

### *tatn-1* acts by *daf-16/*FOXO dependent and independent pathways

**Figure 6:**
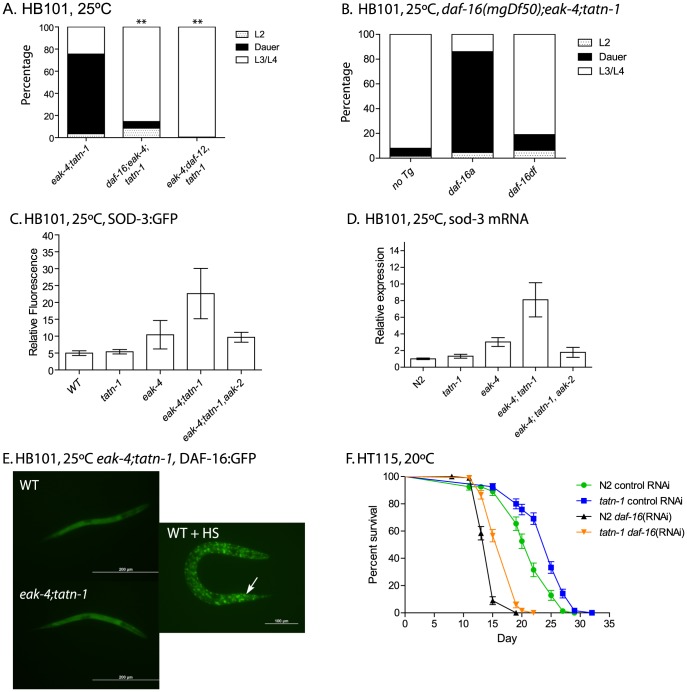
*tatn-1* acts by *daf-16*/FOXO dependent and independent mechanisms. (A) Mutations in *daf-16* (*daf-16(mgDf47)*) or *daf-12* (*daf-12(rh61rh411)*) inhibit dauer formation by *eak-4(mg348); tatn-1(baf1)* mutant worms. ** p<0.001 by Fisher's exact test. (B) The *daf-16(mgDf50)* mutation also inhibits dauer formation by *eak-4; tatn-1(baf1)* mutants, and a transgene expressing the DAF-16A isoform largely rescues dauer formation by *daf-16(mgDf50); eak-4; tatn-1* mutants while a transgene expressing the DAF-16DF isoform only weakly rescues. (C) *eak-4; tatn-1* mutants activate expression of the *daf-16* target gene *sod-3* as indicated by expression of a *sod-3p:GFP* reporter gene in L2 larvae, and this induction requires the *aak-2*/AMPK gene. * p<0.05 for t-test comparisons of *eak-4* vs. *eak-4; tatn-1*, and *eak-4; tatn-1* vs. *eak-4; tatn-1; aak-2*. (D) Induction of the endogenous *sod-3* gene in *eak-4*; *tatn-1* mutants is seen by quantitative RT-PCR, and this induction also depends on the *aak-2*/AMPK gene. (E) DAF-16A:GFP is not nuclear localized in either L2 stage WT or *eak-4; tatn-1* larvae, but exposure to a 1 hour 35°C heat shock produces clear nuclear localization of DAF-16A:GFP (arrow). (F) *tatn-1* effects on lifespan are *daf-16* independent. Treatment of N2 and *tatn-1(baf1)* mutants with *daf-16* RNAi reduces mean survival, but *tatn-1(baf1)* still lives longer than N2. The mean survival of N2 control RNAi, *tatn-1* control RNAi, N2 *daf-16(RNAi)*, *tatn-1 daf-16(RNAi)* are 21.0, 24.0, 14.5, and 17.0 days. p<0.001 for comparison of N2 control RNAi vs. *tatn-1* control RNAi, and *tatn-1 daf-16 (RNAi)* vs. *tatn-1* control RNAi by log-rank test.

Since the effects of *eak* genes on dauer formation are dependent on both the FOXO transcription factor DAF-16 and the nuclear hormone receptor DAF-12, we tested whether these genes are required for dauer formation by *eak-4(mg348); tatn-1(baf1)* mutants through the construction of *daf-16(mgDf47); eak-4(mg348); tatn-1(baf1)* and *eak-4(mg348); tatn-1(baf1), daf-12(rh61rh411)* mutants [26]–[28]. We found that the genetic interaction is completely dependent on *daf-12* (Figure 6A). However, we identified both *daf-16* dependent and independent effects of *tatn-1*. The *tatn-1* effects on dauer formation are largely blocked by the *daf-16(mgDf47)* mutation, but a small percentage of worms still form dauers (Figure 6A). This *daf-16* independent pathway was also seen in experiments using the stronger *tatn-1(qd182)* allele (Figure S6). These findings suggest that *daf-16*/FOXO does play a vital role in the interaction between *eak* genes and *tatn-1*, but that *daf-16*/FOXO independent pathways are also involved.

The *daf-16*/FOXO gene encodes multiple isoforms which have been recently shown to have differential modes of regulation and have distinct effects on development and longevity [24], [55], [56]. For dauer development, three isoforms appear to be involved with DAF-16A being the predominant isoform, DAF-16DF playing a somewhat lesser role, and DAF-16B playing a modest role, at best [55]. As a result, we tested whether the developmental effects of *eak-4* and *tatn-1* are dependent on particular *daf-16* isoforms through the use of isoform-specific transgenes to rescue the dauer-constitutive phenotypes lost in *daf-16*/FOXO mutants (Figure 6B). We found that a transgene encoding a DAF-16A:mRFP fusion protein was able to strongly rescue the formation of dauers by a *daf-16(mgDf50); eak-4(mg348); tatn-1(baf1)* mutant, whereas a transgene encoding a DAF-16DF:GFP fusion protein only weakly rescued dauer formation (Figure 6B). These data suggest that the *daf-16a* isoform is most involved in the developmental effects produced by *tatn-1* mutants.

To further explore the role of *daf-16*/FOXO in *tatn-1* phenotypes, we examined the effects of *eak-4* and *tatn-1* mutations on both *daf-16/*FOXO target gene expression and DAF-16 subcellular localization. We used a *sod-3:GFP* transgene to examine the effects of *eak-4* and *tatn-1* mutations on expression of the *daf-16* target gene *sod-3*, which encodes a manganese superoxide dismutase enzyme [57], [58]. We generated combinations of *eak-4(mg348)* and *tatn-1(baf1)* with the transgene, and examined GFP expression in L2 larvae. We found that the combination of *eak-4* and *tatn-1* mutations resulted in the highest expression of *sod-3:GFP* even prior to dauer formation (Figure 6C). Furthermore, *aak-2*/AMPK was required for this enhanced activation of the *sod-3:GFP* reporter (Figure 6C). Similar effects of *tatn-1* and *eak-4* on *sod-3* expression were also seen when mRNA levels for the endogenous gene were measured by Q-PCR in L2 larvae, and this effect on *sod-3* expression also required *aak-2*/AMPK (Figure 6D). Together these findings demonstrate that *tatn-1* and *eak-4* act in an *aak-2*/AMPK dependent manner to promote at least some aspects of *daf-16*/FOXO transcriptional activity.

Since the *eak* genes act to inhibit DAF-16/FOXO within the nucleus, a possible explanation for the enhancement of dauer formation in *eak; tatn-1* double mutants may be explained by the *tatn-1* allele causing increased nuclear localization of DAF-16 as do mutations affecting *akt-1* [26]. We tested for changes in DAF-16 localization via the use of transgenic animals expressing a DAF-16A:GFP fusion protein [59]. We found that *eak-4; tatn-1* mutants failed to show clearly visible nuclear localization of DAF-16:GFP in synchronized L2 worms grown at 25°C (Figure 6E). In contrast, exposure of animals to a 1 hour heat-shock at 35°C led to strong nuclear localization of DAF-16A:GFP (Figure 6E). Together these findings suggest that the *tatn-1* enhancement of the *eak* dauer formation phenotype could be due to an increase in *daf-16* transcriptional activity without an accompanying significant change in DAF-16 subcellular localization.

We then used *daf-16* RNAi treatment to test whether *daf-16/*FOXO is required for the lifespan extension of *tatn-1* mutants. We found that silencing of *daf-16* through RNAi results in a shortened lifespan for both *tatn-1* and wild-type N2 worms compared to control RNAi treatment (Figure 6F). However in *daf-16* RNAi treated worms, *tatn-1* still produces lifespan extension over wild type (mean survival for N2 control RNAi 21.0 days, *tatn-1(baf1)* control RNAi 24.0 days, N2 *daf-16(RNAi)* 14.5 days, and *tatn-1(baf1) daf-16(RNAi)* 17.0 days). Specifically, *tatn-1(baf1)* produced a 13.7% increase in lifespan in control RNAi treated worms, but a 17.8% increase in lifespan in *daf*-16 RNAi treated worms. These data suggest that the increased lifespan resulting from *tatn-1* is either independent of *daf-16* or occurs in a site resistant to the effects of *daf-16* RNAi.

### *crh-1*/CREB shows overlapping gene expression and phenotypes with *tatn-1*

To explore the effects of impaired tyrosine metabolism in *C. elegans*, we performed whole transcriptome RNA sequencing (RNA-seq) to identify genes that are differentially regulated in the *tatn-1* mutants. To maximize the gene expression changes seen, we used the stronger *tatn-1(qd182)* allele and compared its transcriptome to that of wild-type N2 animals. Using ANOVA testing with a false-discovery rate of 5%, we identified 890 up-regulated and 3732 down-regulated genes in the *tatn-1(qd182)* mutant relative to N2 (Table S4).

To understand how the *tatn-1* mutants might affect the *daf-2*/IGFR pathway, we used this data set to examine whether changes in the expression of genes in pathway are seen. We found that there was no change in the expression of *daf-2*/IGFR, *daf-16*/FOXO, *aak-2*/AMPK, *eak-4*, or *akt-2* (Table S4). However we did find that levels of the *age-1* PI3-kinase are reduced almost 76% and levels of *akt-1* are reduced almost 70% compared to N2 while levels of the *daf-18*/PTEN tumor suppressor, which normally inhibits signaling through the PI3-kinase signaling pathway, is reduced almost 95% compared to wild-type animals (Table S4). Despite the observed changes in the expression of genes in the PI3-kinase signaling pathway, there is likely little net effect on the regulation of downstream targets by the pathway as we failed to observe differences in DAF-16:GFP localization, which would translocate to the nucleus if the pathway was inhibited (Figure 6E).

We then used both the DAVID program and the Panther database both to identify biologic themes within the up-regulated and down-regulated genes by testing for over-represented gene classes based on structural and functional annotations and to visualize the gene classes seen in both groups of genes (Figure S7 and Table S5) [60], [61]. Within the up-regulated set, we found that genes involved in tyrosine metabolism and neuropeptide signaling were strongly over-represented (7–10 fold) (Table S5). Specifically, we found that every gene in the tyrosine degradation pathway is up-regulated in the *tatn-1(qd182)* mutant suggesting that the altered metabolism is detected and leads to a compensatory change in expression of the pathway (Table S4). The significance of the changes in neuropeptide signaling gene expression is currently unclear, but could suggest that impaired tyrosine metabolism or the resulting increased in AAK-2/AMPK activity produces direct changes in neuronal activity or that these changes could be the direct downstream effectors responsible for the *tatn-1* phenotypes. Graphically, we saw a greater percentage of genes which were expressed in the extracellular compartment and had catalytic or receptor activity among the up-regulated genes compared to those that were down-regulated in the *tatn-1* mutants (Figure S7). In contrast, within the down-regulated genes, we identified over-representation of a broad range of genes involved in germline development, cell cycle, DNA replication, and larval development (Table S5). Further, these genes were more likely to be expressed in the intracellular compartment and to have regulatory effects on translation or enzyme activity (Figure S7). The expression changes in genes involved in cell cycle regulation and development could perhaps account for the developmental delay observed in the *tatn-1(qd182)* mutant compared to N2 (Figure S4).

**Figure 7:**
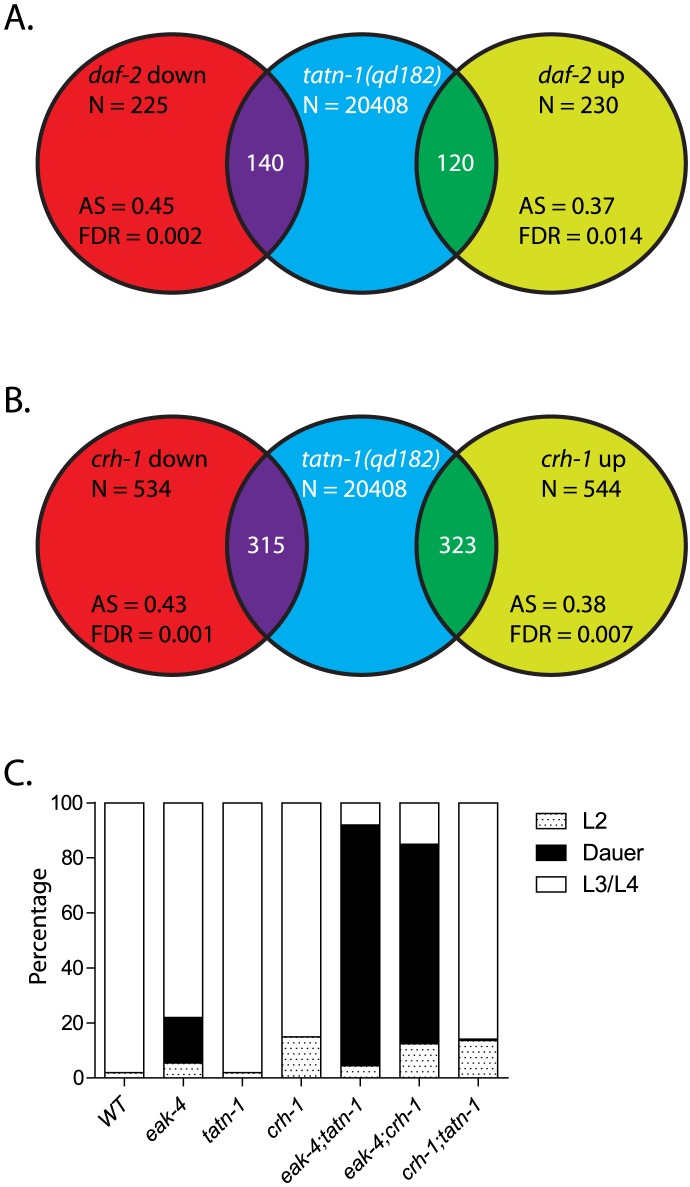
*tatn-1* mutants and *crh-1*/CREB mutants share gene expression profiles and effects on dauer formation. (A) Shot-gun whole transcriptome sequencing (RNA seq) was used to used to characterize and measure the transcriptome of N2 and *tatn-1(qd182)* mutants. From these experiments a total of 20,408 mRNA and other RNA transcripts were detected. To test for evidence of a *daf-16*/FOXO gene expression signature in the *tatn-1(qd182)* mutants Gene Set Association Analysis (GSAA) was used to determine whether the expression of *daf-16*/FOXO target genes regulated in the context of *daf-2/*IGFR signaling are associated with the *tatn-1(qd182)* mutant gene expression profile. GSAA calculates a differential expression score for each gene in the entire 20,408 gene RNA-seq dataset, and then uses a running weighted Kolmogorov-Smirnov test to examine association of an entire gene set with each phenotypic class. The strength of the association is measured by the association score (AS) where positive scores indicate association of the gene set with the phenotype, and statistical significance is measured by a false discovery rate (FDR) that is adjusted for multiple testing. From 225 genes down-regulated in *daf-2*/IGFR mutants, 140 showed association with the *tatn-1(qd182)* profile, and from 230 genes up-regulated in *daf-2*/IGFR mutants, 120 showed evidence of association by GSAA analysis. AS represents the association score with positive values indicating association, and FDR represents the false discovery rate for the association. (B) To test for evidence of a *crh-1*/CREB gene expression signature in the *tatn-1(qd182)* mutants Gene Set Association Analysis (GSAA) was used to determine whether the expression of *crh-1*/CREB target genes identified through microarray studies using wild-type and *crh-1* mutant worms associate with the *tatn-1(qd182)* expression profile. From 534 genes down-regulated in *crh-1*/CREB mutants, 315 showed evidence of association, and from 544 genes up-regulated in *crh-1*/CREB mutants, 323 showed evidence of association by GSAA analysis. AS represents the association score with positive values indicating association, and FDR represents the false discovery rate for the association. (C) *crh-1*/CREB mutants enhance dauer formation by *eak-4* mutants but not by *tatn-1* mutants.

Given the requirement we found for *daf-16*/FOXO for aspects of the *tatn-1* phenotypes, we used Gene Set Association Analysis (GSAA) to test for enrichment of genes known to be regulated by *daf-16*/FOXO in the context of *daf-2*/IGFR signaling [62], [63]. Via this approach, we found both the up-regulated and down-regulated *daf-16*/FOXO target genes identified by Murphy et. al. to be enriched within the *tatn-1(qd182)* transcriptome (Figure 7A). This suggests that the expression of a subset of *daf-16*/FOXO target genes is altered by changes in tyrosine metabolism.

Since our genetic studies suggested the involvement of a *daf-16*-independent pathway, we also used GSAA to test whether target genes recently identified for the CREB transcription factor *crh-1* are enriched in the *tatn-1(qd182)* mutant [53]. We chose to focus on *crh-1* because recent work has demonstrated that *crh-1*/CREB lies downstream of *aak-2*/AMPK [53]. *crh-1*/CREB and *aak-2/*AMPK are mechanistically linked because AAK-2 directly phosphorylates and inactivates the *crh-1*/CREB coactivator *crtc-1*, and as a result both *aak-2*/AMPK over-expressing and *crh-1*/CREB mutant animals are long-lived and share gene expression profiles [53]. We found that in the *tatn-1(qd182)* mutants, there is differential expression of both genes up-regulated and genes down-regulated in *crh-1*/CREB mutants (Figure 7B). This suggests that altered tyrosine metabolism could lead to changes in *crh-1* target gene expression and could suggest a role for *crh-1*/CREB in the *tatn-1* phenotypes. To test for *crh-1*/CREB involvement, we combined the *crh-1(tz2)* null allele with *tatn-1* and *eak-4* and examined the effects on dauer formation. We found that *crh-1* showed a similar interaction as *tatn-1* with *eak-4*, but did not promote dauer formation by the *tatn-1* mutant (Figure 7C). Together these findings suggest that *tatn-1* mutants share phenotypes and gene expression profiles with *crh-1*/CREB mutants and could be consistent with *crh-1*/CREB acting as an additional downstream effector of the response to impaired tyrosine metabolism.

### Tyrosine aminotransferase expression is controlled by diet and environment

**Figure 8:**
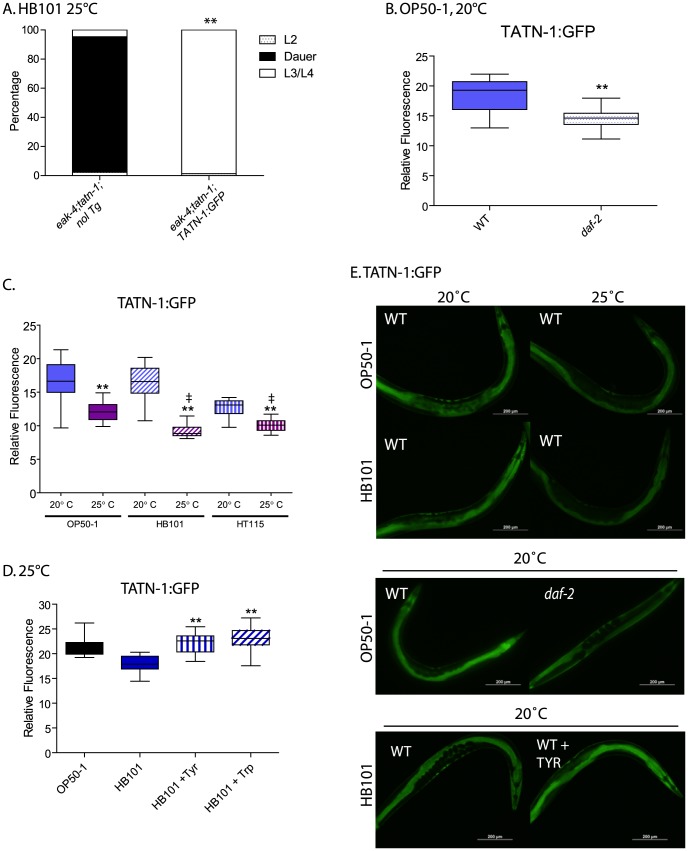
Control of TATN-1 protein expression by *daf-2*/IGFR signaling, diet and temperature. (A) Rescue of *tatn-1* mediated dauer arrest by a *tatn-1p:tatn-1 cDNA:GFP* transgene (*bafIs131)*. (B) *daf-2* signaling positively regulates *TATN-1:GFP* expression. Box and whiskers plot showing comparison of GFP fluorescence between day 1 adult wild-type and *daf-2(e1368)* mutants expressing the *TATN-1:GFP* transgene and grown on OP50-1 bacteria. N≥15 worms, ** p<0.001 by t-test. (C) Bacterial food source and temperature regulate *TATN-1:GFP* expression. Wild-type worms expressing the *TATN-1:GFP* transgene were grown on the indicated bacteria until adulthood and then either kept at 20°C or shifted to 25°C overnight. N≥15 worms, ** p<0.001 for 20°C versus 25°C for each bacterial strain by t-test, and ‡ p<0.001 for 25°C treatment of OP50-1 versus HB101 or HT115. (D) Supplementation of HB101 with 1 mg/mL tyrosine or tryptophan increases *TATN-1:GFP* expression. Wild type worms expressing the *TATN-1:GFP* transgene were grown until adulthood on the indicated diet. N≥15 worms, ** p<0.001 for comparison between HB101, and HB101+Tyr or HB101+Trp by t-test. (E) Representative photos showing the effects of the treatments graphed in panels B-D.

In vertebrates, tyrosine aminotransferase has been reported to be an insulin target gene with insulin treatment leading to reduced expression [32]–[35], [38]. As a result, we asked whether *tatn-1* could also be regulated by *daf-2*/IGFR signaling in worms. To test the effects of *daf-2* signaling on *tatn-1* expression, we generated transgenic worms with an integrated transgene expressing a TATN-1:GFP fusion protein under the control of the *tatn-1* promoter. This transgene rescues the *tatn-1(baf1)* mutation and blocks dauer formation by *eak-4(mg348); tatn-1(baf-1)* mutants, which demonstrates that the fusion protein is both functional and expressed in the correct anatomical locations (Figure 8A).

GFP fluorescence representing the TATN-1:GFP fusion protein is observed in the intestine and hypodermis of worms (Figure 8E). When we crossed the transgene into the *daf-2(e1368)* mutant, and we found that the presence of the *daf-2*/IGFR mutation led to a 20% decline in GFP expression in adult worms grown on OP50-1 at 20° (Figure 8B and Figure 8E). This is consistent with *daf*-2/IGFR signaling acting positively to promote TATN-1 expression in adult worms. Interestingly, the effect of *daf-2/*IGFR signaling on TATN-1 expression likely occurs either at the translational or protein stability level because Q-PCR experiments demonstrated almost a 50% increase in *tatn-1* mRNA expression in the *daf-2* mutant animals (Figure S8). As a result of the divergent regulation of *tatn-1* mRNA and protein levels, we focused on TATN-1:GFP expression in our subsequent experiments.

Beyond *daf-2*/IGFR signaling, we found that both diet and environmental temperature affected TATN-1 levels to a similar or even greater degree. Specifically, adult worms grown on OP50-1, HB101, or HT115 show decreases in TATN-1:GFP expression when shifted from 20°C to 25°C for 24 hours with worms grown on OP50-1 showing a 26.2% decrease, on HB101 a 44.7% decrease, and on HT115 a 24.2% decrease (Figure 8C and Figure 8E). Further, we found that the OP50-1 fed worms showed greater TATN-1:GFP expression compared to HB101 and HT115 fed worms. This difference was especially apparent in worms grown at 25°C, due to the variability of GFP intensity seen at 20°C, with HB101 and HT115 fed animals showing a 25.3% and 17.3% decrease, respectively, compared to OP50-1 fed worms (Figure 8C and Figure 8E).

The effects of diet on TATN-1:GFP expression suggests that the *E. coli* bacterial strains vary in nutrient composition in a way that can be detected by the worms. Prior work has demonstrated that protein is the primary component of these bacteria but that the overall protein levels are not significantly different between strains [64]. However, this work also suggested that specific amino acids could vary between the strains and account for differences in fat content in worms fed each strain. Specifically, *pept-1* mutants, which lack an intestinal peptide transporter, fail to show the expected differences in fat content when fed different bacterial strains [64]. In vertebrates, tyrosine aminotransferase expression is controlled by dietary amino acid intake, most notably for tryptophan [65]–[67]. To test whether dietary amino acid intake could affect TATN-1:GFP expression, we supplemented HB101 spotted NGA plates spotted with either tyrosine or tryptophan at a final concentration of 1 mg/mL, and compared TATN-1:GFP expression to worms fed HB101 alone or OP50-1. This concentration is 8 times the level found in standard NGA media (0.125 mg/mL). We found that the addition of tyrosine or tryptophan increases the GFP expression level in HB101 fed worms up to that seen in worms grown on OP50-1 (Figure 8D and Figure 8E). Together these data demonstrate that TATN-1 levels are dynamic and under the control of both *daf-2*/IGFR signaling as well as dietary and environmental cues. Importantly many of these signals that control TATN-1 expression also influence dauer formation suggesting that *tatn-1* could be a regulated modulator of *daf-2*/IGFR signaling and developmental decisions.

### Tyrosine mediates the effects of *tatn-1* mutations

**Figure 9:**
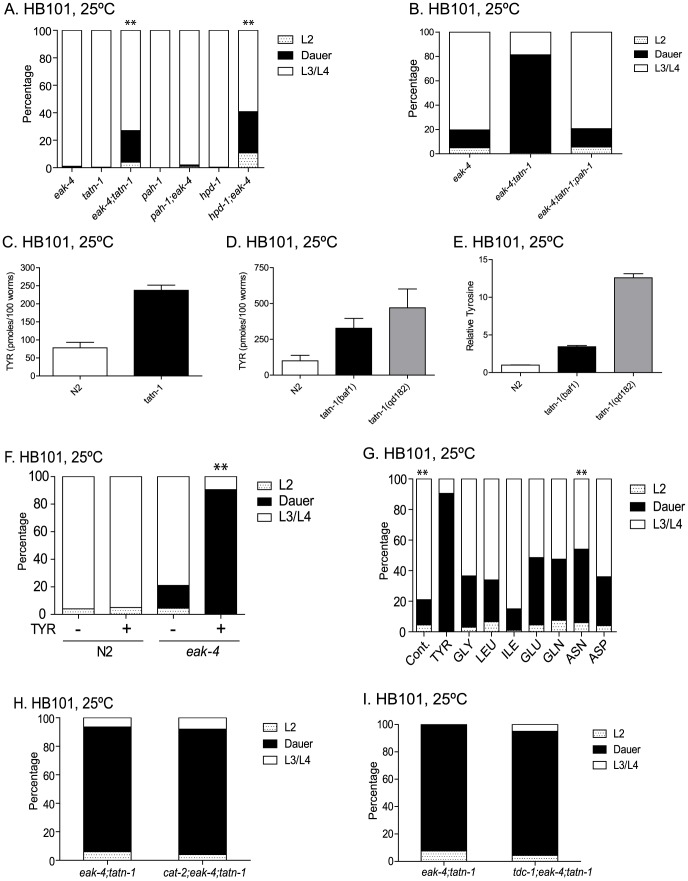
*tatn-1* acts by increasing tyrosine levels. (A) Both *tatn-1* and *hpd-1* mutations augment dauer arrest by *eak-4(mg348)* mutants after three days at 25°C, whereas a *pah-1* mutation does not. ** p<0.001 for comparisons between *eak-4* and *eak-4; tatn-1*, and *hpd-1; eak-4* by Fisher's exact test. (B) Inhibiting tyrosine synthesis with the *pah-1* mutation reduces dauer formation by *eak-4*; *tatn-1* mutants. (C) *tatn-1(baf1)* has a higher concentration of tyrosine compared to wild type N2. The bars represent the mean tyrosine concentration from four independent samples, and the error bars show the standard error of the mean. ** p<0.001 by t-test. (D) *tatn-1(qd182)* and *tatn-1(baf1)* have higher tyrosine levels compared to wild type N2. (E) *tatn-1(qd182)* has higher tyrosine levels than N2 and *tatn-1(baf1)* when the measured tyrosine levels are normalized to the levels of non-aromatic amino acids in each sample. (F) Treatment of *eak-4(mg348)* mutants but not N2 with tyrosine results in dauer arrest. ** p<0.001 for comparison of *eak-4* + and - tyrosine using Fisher's exact test. (G) Tyrosine (1 mg/mL) is more potent than other amino acids in producing dauer arrest by *eak-4(mg348)* mutants. ** p<0.001 for comparison between *eak-4* vs *eak-4*+tyrosine and *eak-4*+tyrosine and *eak-4*+asparagine using Fisher's exact test. (H) Impaired dopamine synthesis in *cat-2(e1112)* mutants does not block the effect of *tatn-1* on dauer formation. (I) Impaired tyramine and octopamine synthesis in *tdc-1(ok914)* mutants does not block the effect of *tatn-1* on dauer formation.

Since TATN-1 is the first enzyme in the tyrosine degradation pathway, decreased activity should both increase the levels of tyrosine and also decrease the levels of the downstream metabolites (Figure 1B). The *tatn-1* mutant phenotypes could be a direct result of changes in the level of a particular metabolite. For example, elevated fumarate levels are known promote hypoxia inducible factor (HIF) activity in certain renal cancers [68]. Alternately, *tatn-1* could have a novel function that is independent of its metabolic activity. For example, subunits of the phenylalanine hydroxylase enzyme are known to have an additional, non-enzymatic role as a transcriptional co-activator [69]. To explore whether either of these models accounts for the *tatn-1* phenotypes, we tested for interactions between *eak-4* and mutant alleles of other enzymes in the tyrosine degradation pathway (Figure 1B) by constructing *pah-1; eak-4* and *hpd-1; eak-4* mutants. These mutants lack *pah-1*, which encodes the enzyme phenylalanine-4-hydroxylase that converts phenylalanine into tyrosine, or *hpd-1*, which encodes 4-hydroxyphenylpyruvate dioxygenase and catalyzes the step immediately downstream of *tatn-1* [39], [43], [70]. We found that *pah-1* did not enhance dauer arrest by the *eak-4* mutants (Figure 9A) whereas both *tatn-1* and *hpd-1* increased dauer formation. Lower numbers of *eak-4; tatn-1* dauers were seen because the experiment was scored after 3 days due to the slower development of the *hpd-1* mutant. Additionally, when we used the *pah-1* mutant to block the synthesis of tyrosine in a *tatn-1; eak-4* mutant, we found that this reduced dauer formation (Figure 9B). These results suggested that the effects of *tatn-1* on dauer formation were directly linked to the metabolic effects of *tatn-1*, and that the accumulation of tyrosine instead of deficiency of a downstream metabolite could be responsible for the *tatn-1* phenotype.

As a result, we measured the levels of amino acids in wild-type N2, *tatn-1(baf1)*, and *tatn-1(qd182)* larval animals grown at 25°C on HB101 plates by liquid chromatography mass spectrometry (LC-MS/MS). We found that wild-type N2 worms contained an average of 78.1 pmol of tyrosine per 100 worms whereas *tatn-1(baf1)* worms contained 237.4 pmol per 100 worms (Figure 9C). Hence the *tatn-1(baf1)* mutation produced a roughly three fold increase in tyrosine levels in the mutant animals. Further to compare the effects of *tatn-1(qd182)* on tyrosine levels compared to *tatn-1(baf1)*, we measured tyrosine levels in additional samples grown and prepared in parallel. We found in these samples that wild-type N2 worms contained an average of 99.6 pmol of tyrosine per 100 worms, *tatn-1(baf1)* contained an average of 327.2 pmol per 100 worms, and *tatn-1(qd182)* contained an average of 470.2 pmol per 100 worms, which is an 43.7% increase over *tatn-1(baf1)* (Figure 9D). However, we noted that the *tatn-1(qd182)* worms were smaller than N2 or *tatn-1(baf1)* and the levels of many other amino acids measured in parallel were lower in *tatn-1(qd182)* compared to *tatn-1(baf1)*. This suggested that the *tatn-1(qd182)* samples may have contained less overall biomass, and hence our normalization to worm counts alone may underestimate the effect of the *tatn-1(qd182)* mutation on tyrosine levels. To correct for this difference, we normalized tyrosine levels to the levels of all non-aromatic amino acids in the samples with the assumption that the net effect of these mutations on the levels of these amino acids is neutral. After normalization, we found that *tatn-1(baf1)* produced a 3.4 fold increase in tyrosine levels compared to N2 whereas *tatn-1(qd182)* produced a 12.6 fold increase compared to N2, which is also a 3.7 fold increase over *tatn-1(baf1)* levels (Figure 9E). These findings demonstrate that both *tatn-1* alleles increase tyrosine levels compared to wild-type animals, and that the stronger phenotypes of the *tatn-1(qd182)* allele are likely due to the further increases in tyrosine levels observed.

To directly test whether elevated tyrosine levels are responsible for the *tatn-1* phenotype, we treated worms with exogenous tyrosine cast into the NGA plates at 1 mg/mL. This treatment results in tyrosine levels in the worms that are elevated compared to untreated animals, but lower than those seen in the *tatn-1* mutants (Figure S9). We found that supplementation had no effect on the development of wild-type worms but lead to dauer arrest by the *eak-4* mutants (Figure 9F). These results directly demonstrate changes in tyrosine levels alter the development of *eak-4* mutant worms and are responsible for the *tatn-1* phenotype.

Amino acids are known to antagonize insulin actions in vertebrates, so our results could represent either the non-specific effects of any amino acid or a tyrosine-specific effect [71], [72]. To test these possibilities we directly compared the ability of a variety of amino acids to enhance dauer formation by *eak-4* mutants. We grew *eak-4* mutants on HB101 spotted NGA supplemented with tyrosine, glycine, leucine, isoleucine, glutamate, glutamine, asparagine, or aspartate, each at the concentration of 1 mg/mL. Since tyrosine is the largest of these amino acids, this resulted in worms being treated with higher molar equivalents of the other amino acids compared to tyrosine. We found that while other amino acids do increase the formation of dauers by *eak-4* mutants, none was as potent as tyrosine (Figure 9G). This suggests that the effect on dauer formation shows selectivity for the presence of tyrosine. The effects of tyrosine and to a lesser extent the other amino acids is not due to a toxic effect of the amino acid supplementation as treated worms showed a similar lifespan to untreated worms (Figure S9).

Besides being a building block for proteins, tyrosine serves as a precursor for the synthesis of catecholamine neurotransmitters. In vertebrates, there is evidence that the levels of tyrosine as a precursor influences the synthesis of these neurotransmitters [73]. Hence, one possibility is that *tatn-1* mutations raise tyrosine levels and facilitate its conversion into the neurotransmitters dopamine, octopamine, or tyramine which could produce the observed phenotypes. To test this possibility, we blocked dopamine synthesis with the *cat-2* mutation, which affects the worm tyrosine hydroxylase gene, and we blocked octopamine and tyramine synthesis with the *tdc-1* mutation, which removes the enzyme tyrosine decarboxylase [74], [75]. We found that both *cat-2; eak-4; tatn-1* and *tdc-1; eak-4; tatn-1* mutants are similar to *eak-4; tatn-1* mutants with regards to the formation of dauers (Figure 9H and Figure 9I). These data demonstrate that excessive synthesis of dopamine, octopamine, or tyramine is not responsible for the *tatn-1* phenotype. Instead tyrosine is directly sensed by the worms and acts as a developmental regulator.

## Discussion

### Tyrosine as a modulator of *daf-2* insulin/IGF-1 signaling effects

**Figure 10:**
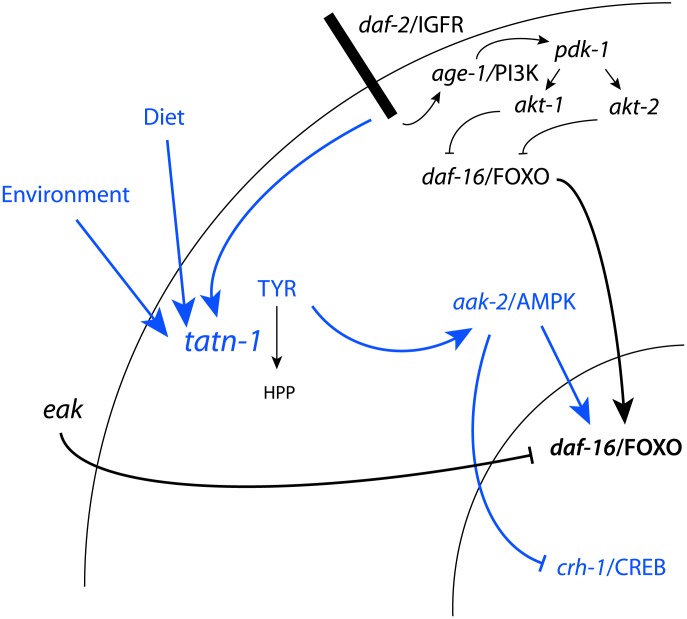
Model for the regulation of TATN-1 expression, tyrosine levels, and the resulting effects of tyrosine effects on cell signaling pathways. Shown is the *daf-2*/IGFR signaling pathway and *eak* genes from Figure 1A with the addition, shown in blue, of the effects of tyrosine identified in this work and the control of TATN-1 expression by *daf-2*/IGFR signaling and by dietary and environmental cues as demonstrated in Figure 8. In summary, worms sense the available diet and environmental temperature and these factors both contribute to the dauer decision and the regulation of TATN-1 protein levels. Conditions which promote the dauer decision also tend to reduce the expression of TATN-1. The reductions in TATN-1 expression lead to reduced removal of tyrosine through degradation and increase free tyrosine levels in the animal. These increases in tyrosine activate *aak-2*/AMPK and disrupt the effects of normal *daf-2*/IGFR signaling through positive effects on *daf-16*/FOXO and perhaps inhibitory effects on *crh-1*/CREB. The effects of tyrosine are particularly pronounced when the *daf-2*/IGFR pathway is compromised, such as through mutations in the *daf-2*/IGFR gene or the *eak* genes which lie in a parallel pathway. This may be due to further reductions in TATN-1 expression or reductions in inhibitory regulators of *daf-16*/FOXO activity.

Together our results identify tyrosine and tyrosine aminotransferase activity as a modifier of *daf-2*/IGFR effects in *C. elegans* (Figure 10). While the control of tyrosine aminotransferase expression and activity has been extensively studied as a target of insulin signaling in vertebrates [30]–[34], [36], [38], a connection between tyrosine aminotransferase or tyrosine metabolism and insulin action has not been demonstrated. Prior work in *C. elegans* has suggested that the *hpd-1* gene, which encodes 4-hydroxyphenylpyruvate dioxygenase, is repressed in *daf-2* mutants and that knock-down of *hpd-1* by RNAi delayed dauer exit and extended lifespan [39]. However, the mechanism involved has been unclear. Our work shows that both *tatn-1*, and likely *hpd-1*, impact on *daf-2*/IGFR signaling through increasing tyrosine levels in the animal.

We find the effects of tyrosine on *daf-2*/IGFR signaling to be complex with roles for both the *daf-16*/FOXO transcription factor and the *aak-2*/AMPK seen (Figure 10). One way that high tyrosine levels could interact with *daf-2*/IGFR signaling would be for tyrosine to somehow activate *aak-2*/AMPK. AMP kinases are a known regulator of both *daf-16* and the vertebrate homolog FOXO3 [51], [76]. AMPK regulates FOXO transcriptional activity through the phosphorylation of up to six sites on these proteins. In our work, *aak-2*/AMPK mutations suppress the dauer promoting effects of *tatn-1* mutations, treatment of worms with the AMPK agonist AICAR is able to mimic the effects of tyrosine, and increases in the active phosphorylated form of AAK-2 are seen in the *tatn-1* mutant. These findings demonstrate that elevated tyrosine levels activate *aak-2*/AMPK which could then phosphorylate *daf-16*/FOXO. This phosphorylation event could then interfere with the inhibitory effects of an intact *daf-2*/IGFR pathway on *daf-16*/FOXO activity. Further work would be needed to test this model, and the ability of mutants lacking *aak-2*/AMPK or *daf-16*/FOXO to still respond to elevated tyrosine levels also supports the presence of alternate, currently unknown downstream pathways.

These alternate pathways could either lie in parallel to *daf-16*/FOXO or could be the dominant response pathway with *daf-16*/FOXO only playing a permissive role, especially at lower tyrosine levels. One possible alternate pathway involves the CREB transcription factor *crh-1* (Figure 10). The *crtc-1* co-activator for the CREB transcription factors has been shown to be a target of regulation by *aak-2*/AMPK, and in vertebrates, CRTC co-activators are known to interact with insulin signaling in mediating the hepatic metabolic adaptation to the fed versus fasting state [53], [77], [78]. We find that *tatn-1(qd182)* mutants show evidence of *crh-1*/CREB-regulated gene expression, and a *crh-1*/CREB mutant mimics the interaction of *eak-4* and *tatn-1*. Perhaps elevated tyrosine levels lead to the activation of *aak-2*/AMPK which then results in the activation of *daf-16*/FOXO and inhibition of *crtc-1* and *crh-1*/CREB (Figure 10). The presence of paired downstream pathways could explain the partial requirement for *daf-16*/FOXO, especially at higher tyrosine levels.

Beyond effects on *daf-16*/FOXO and *crh-1*/CREB, elevated tyrosine, especially at high levels, could also have hormetic effects via changing cellular redox status, producing ER stress, or perturbing the protein folding environment [79]. These effects could account for the positive effects of increased tyrosine on longevity, and some of the genes involved in sensing hormetic stresses, such as the HSF-1 ortholog *hsf-1*, also interact with *daf-16*/FOXO and play roles in dauer formation [80]–[82]. Alternately, tyrosine could act via a novel pathway that operates independently of *daf-16*/FOXO or *crh-1*/CREB, especially at higher levels. For example, the vertebrate calcium-sensing receptor, PPARγ nuclear receptor, and aryl hydrocarbon receptor (AHR) have all been shown to respond to aromatic amino acids, though their connection to insulin action is currently unclear [83]–[85]. The recent finding that Akt and Foxo1 are largely dispensable for the control of hepatic metabolism by insulin in vivo has suggested that FOXO- independent pathways exist and play important roles in metabolic control [86].

Our work also provides insights into the *eak* genes which are known to act via unclear mechanisms to reduce *daf-16*/FOXO transcriptional activity while not significantly affecting the subcellular localization of DAF-16/FOXO [26], [27], [29]. We find that beyond enhancing the inhibitory effects of *akt-1* on *daf-16*/FOXO activity, the *eak* genes also suppress the effects of amino acids and AMPK activity on *daf-16*/FOXO activity. Additional work will be needed to understand if the *eak* genes normally represent a control point where the effects of these metabolic signals on insulin signaling can be enhanced or suppressed.

### The complex control of tyrosine aminotransferase

We find that the regulation of *tatn-1* expression in worms is complex with *daf-2* activity, diet, and environmental conditions each contributing to the expression level (Figure 10). In vertebrates, tyrosine aminotransferase has also been shown to undergo regulation at the transcriptional, translation, and degradation levels in response to hormonal and nutritional cues [66]. We find that *daf-2*/IGFR activity inhibits *tatn-1* gene transcription but raises TATN-1 protein levels. This is consistent with work in vertebrates showing that insulin shows complex effects on tyrosine aminotransferase expression with actions at both the transcriptional and translational level [30]–[34], [36], [38]. Nutritional cues appear to also be an important regulator because we find that the *E. coli* strain used as food has a powerful effect on the expression of *tatn-1* and these effects parallel the effects of the weaker *tatn-1(baf1)* allele on dauer formation. In rats, the activity of hepatic tyrosine aminotransferase varies several-fold during the day with a peak during the evening and nadir in the early morning [87]. Studies of the cyclic variation have demonstrated that dietary protein intake is a prime inducer of tyrosine aminotransferase levels [65]. Feeding animals a protein-free diet results in a constant low level of tyrosine aminotransferase, whereas feeding animals protein meals at differing times produces corresponding shifts in enzyme production. Among amino acids, some such as tryptophan are potent inducers of tyrosine aminotransferase expression [66]. The mechanisms accounting for the dietary effects of amino acids on tyrosine aminotransferase are currently unclear. This could suggest a role for additional nutrient sensitive pathways which may well be conserved as we find that both tryptophan and tyrosine act as *tatn-1* inducers in worms. Finally, we find a novel role for environmental conditions on *tatn-1* expression as lower temperatures promote expression and higher temperatures inhibit it. How changes in temperature translate into the observed effects is unclear but perhaps hormonal changes mediated by the cytochrome P450 *daf-9* and the nuclear hormone receptor *daf-12* or changes mediated by thermosensory neurons are involved [88]. Together this suggests that the control of tyrosine aminotransferase activity, which modulates tyrosine levels, could be controlled via a complex network of internal and external cues. Our finding that changes in tyrosine levels alter both signaling pathways and gene expression patterns could suggest that carefully controlling tyrosine metabolism and ultimately tyrosine levels plays an important role in overall homeostasis (Figure 10).

### Aromatic amino acids in human disease

Recent work has suggested that levels of specific amino acids, particularly branched chain and aromatic amino acids, could influence insulin sensitivity in people and mice [1]–[6]. While the exact role of aromatic amino acids in metabolic disease is unknown, our results suggest that these could play a causal role in either insulin-resistance or the development of diabetes [89]. Given the complex nature of tyrosine aminotransferase regulation, subtle changes in hormone levels, diet, and perhaps other factors could lead to changes in hepatic tyrosine metabolism and contribute to changes in serum aromatic amino acid levels. There may also be significant changes during the day due to dietary intake or release from internal stores such as muscle. As tyrosine levels increase, it is possible that, as in worms, the increases modify responses to insulin signaling and augment pre-existing insulin resistance in a harmful way (Figure 10). The connection between insulin signaling and tyrosine metabolism could potentially even lead to a vicious cycle of reduced insulin signaling producing elevated tyrosine levels which then lead to a further reduction in insulin signaling

Tyrosine aminotransferase has also been found to be a tumor suppressor gene in human hepatocellular carcinoma (HCC) [7]. The human tyrosine aminotransferase gene is located on 16q, which is frequently deleted in HCC, and analysis of tumors reveals that gene deletion or silencing via hypermethylation is common [7]. Consistent with an inhibitory role in the pathogenesis of liver cancer, transfection of HCC cancer cell lines with a tyrosine aminotransferase transgene suppressed malignant behavior such as growth in soft agar and the formation of tumors in nude mice. In these cells, tyrosine aminotransferase expression also acted to inhibit tumor formation via the stimulation of apoptosis, but the exact molecular events are still unclear [7]. Our data would suggest that the activation of AMPK or the downstream effects of AMPK on FOXO transcription factors, such as FOXO3, or CREB would be attractive targets for future study. Alternately, we also saw down-regulation of genes involved in DNA repair so the elevated tyrosine levels could also promote the accumulation of additional cancer promoting mutations (Table S5). Together these findings suggest that extracellular or intracellular tyrosine levels could act as signaling molecules involved in the control of cell growth, differentiation, and physiology.

## Materials and Methods

### *C. elegans* strains and maintenance

All *C. elegans* strains were propagated on standard nematode growth agar (NGA) plates containing streptomycin (200 µg/mL) and spotted with OP50-1, as previously described [90]. For specific experiments, worms were fed HB101, OP50, or HT115 *E. coli* strains using NGA containing streptomycin (HB101) or no antibiotics (OP50 and HT115).

The following *C. elegans* mutants were obtained from the *C. elegans* Genetics Center, which is supported in part by NIH Office of Research Infrastructure Programs (P40 OD010440): *daf-16(mgDf50)* I, *cat-2(e1112)* II, *pah-1(ok687)* II, *tdc-1(ok914)* II, *crh-1(tz2)* III, *daf-2(e1368)* III, *hpd-1(ok1955)* III, *unc-119(ed3)* III, *akt-1(mg144)* V, *akt-1(mg306)* V, *aak-2(gt33)* X, *akt-2(ok393)* X, *daf-12(rh61rh411)* X, *pdk-1(mg142)* X, *sgk-1(ok538)* X, *muIs84[pAD76(sod-3::GFP)]*, *lpIs14 [daf-16f::GFP+unc-119(+)]*, and *lpIs12 [daf-16a::RFP+unc-119(+)]. tatn-1(baf1)* X has been described previously, and *tatn-1(qd182)* was identified in an unrelated mutagenesis screen and is a gift from Daniel Pagano and Dennis Kim [43], [44]. *muIs109[daf-16::GFP]* X has been described previously and is a gift from Malene Hansen [59]. *daf-16(mgDf47)*, *hsd-1(mg433)* I, *eak-3(mg344)* III, *eak-4(mg348)* IV, *eak-7(tm3188)* IV, *sdf-9(mg337)* V have been described previously [26]–[28], [91]. Double and triple mutants were generated by standard genetic crosses, and the genotypes of strains were confirmed by PCR using oligos which detect gene deletions or RFLP's associated with the mutation (Table S6). Throughout this work *tatn-1* is implied to refer to the *tatn-1(baf1)* allele except specifically as noted otherwise.

### Dauer assays

Worm embryos were isolated by sodium hypochlorite treatment, and eggs were transferred to plates, and grown at the indicated temperature in a designated incubator. The plates were scored two or three days later under a dissecting microscope for the presence of L2, dauer, and L3/L4 and older worms. We conducted control experiments to determine the robustness and reproducibility of visual scoring by having several scorers evaluate a series of still images and corresponding movies of larvae of different developmental stages. We then compared the correlation between the scorers for the entire series via the use of a kappa statistic [92]. These experiments indicated that scoring was consistent between raters within the lab with all comparisons showing “substantial” to “almost perfect” agreement (Table S7) [92].

For each assay, approximately 100 worms were scored from each of two to three plates set up in parallel for each genotype used in an experiment. This resulted in 200 to, more typically, 300 animals being scored for each genotype within an experiment. Each experiment was repeated at least once with comparable results, which resulted in between 400–600 worms being scored per genotype in total. The percentages of each stage were graphed using Prism5 software, and the graphs show pooled data from a single trial. To perform pairwise comparisons between mutant strains, a contingency table was set up using the counts for L2, dauer, and L3/L4 categories, and p-values were calculated using Fisher's exact contingency test within SAS version 9.3. To determine SDS resistance, worms were washed from plates with 1% SDS, and then incubated for 20 minutes with gentle rocking. Worms were then pelleted and washed with water. Aliquots were scored for survival as demonstrated by movement, and each experiment was repeated at least once. The percentages of living worms were graphed with Prism5.

### Lifespan assays

Lifespan assays were conducted as previously described at 20°C using either NGA or RNAi plates containing 50 µM FUDR [93]. Lifespan assays for N2, *tatn-1(baf1)*, *eak-7(tm3188)*, and *eak-7(tm3188); tatn-1(baf1)* used NGA plates spotted with HB101, and worms were grown from eggs at 16°C to minimize larval arrest. Lifespan assays for N2, *tatn-1(baf1)*, *aak-2(gt33)*, and *tatn-1(baf1), aak-2(gt33)* used NGA plates spotted with HB101. Lifespan assays for amino acid treated N2 worms used either NGA plates or NGA plates supplemented with 1 mg./mL tyrosine, glycine, or isoleucine, and then spotted with HB101. Lifespan assays using *daf-2* and *daf-16* RNAi treatment used NGA media supplemented with carbenicillin (50 µg/mL) and isopropyl β-d-thiogalactopyranoside (IPTG, 1 mM). RNAi treatment for *daf-16* was started at egg hatching while *daf-2* RNAi treatment started on day 1 of adulthood with larval development occurring on NGA plates spotted with HB101 at 20°C.

For all lifespan assays three plates containing 40 worms each, for each genotype were set up, as well as an extra plate, with worms to replace worms that had crawled off the plate, bagged, or exploded, to reduce the number of censored events. Prism5 (Graphpad Software) was used to generate graphs and perform log-rank testing for curve comparisons. SAS was used to create lifetables and calculate mean survival.

### Amino acid supplementation

Amino acids (Sigma-Aldrich) were dissolved as 45 mg/mL stock solutions in water, and then added to molten NGM to obtain a 1 mg/mL final concentration. These plates were dried and spotted with HB101 before use.

### Amino acid analysis

Worms were grown on HB101 spotted NGM plates for two days at 25°C before being washed from the plates and then being rinsed twice with miliQ water. For Figure S9A, NGM plates either with or without 1 mg./mL tyrosine cast into the agar were used to grow the worm culture. To normalize the samples for worm number, a 5 µL aliquot was removed and scored for worm number. Amino acids were then extracted using aqueous methanol and crushing with a mortar and pestle as previously described [94]. The methanol solution was removed by evaporation and the residue stored frozen at −80°C. Amino acid analysis was performed via liquid chromatography tandem mass spectrometry following reconstitution of the residue in 0.1 mL water as described previously for urine [95]. Amino acid content was then either normalized to total worm number in the sample (Figure 9B, Figure 9C, and Figure S9A) or normalized to the levels of individual non-aromatic amino acids and then divided by the average normalized level observed in the wild-type N2 samples (Figure 9D).

### AICAR treatment

Aliquots from a 250 mM AICAR solution dissolved in water (Cell Signaling Technology) were spotted onto NGA plates spotted with HB101 to give a final concentration of 0.125 mM 1 hour before eggs were added to the plates. A comparable volume of water alone was used as a negative control.

### AMP and ATP measurements

Worm embryos were isolated by sodium hypochlorite treatment from N2 and *eak-4(mg348); tatn-1(baf1)* adults, and the eggs were transferred to NGA plates spotted with HB101. The plates were incubated at 25°C for 24 hours so most of the population was L2 larvae. The worms were washed from plates with water and washed with water to remove bacteria. Nucleotides were then extracted from the worms as previously described [50]. The resulting extract was stored at −80°C until analysis. ATP, ADP, and AMP levels were measured by HPLC with UV detection of individual nucleotides.

### Fluorescent imaging

Images of worms were obtained with a BX51 fluorescence microscope and quantified using ImageJ software as previously described [44].

### Quantitative RT-PCR

For *sod-3* expression, worm embryos were isolated by sodium hypochlorite treatment, and eggs were transferred to NGA plates spotted with HB101 and incubated at 25°C for 24 hours. The worms were washed from plates in water, pelleted by centrifugation, washed with water, and frozen for storage. RNA extraction, reverse transcription, and quantitative PCR were performed as previously described [27], [44]. The geometric mean level of the control genes *pmp-3, cdc-42*, and *Y45F10D.4* were used to normalize the samples, and the relative levels of *sod-3* expression were determined using the 2^−ΔΔCt^ approach [96], [97].

For *tatn-1* expression, N2 and *daf-2(e1368)* embryos were isolated by sodium hypochlorite treatment, and eggs were transferred to S-basal to arrest the worms at the L1 stage. L1 larvae were added to NGA plates spotted with OP50-1 and the plates were incubated at 20°C for 3 days. Adult worms were washed from plates and RNA was isolated as described above. The geometric mean level of the control genes *pmp-3, cdc-42*, and *Y45F10D.4* were used to normalize the samples, and the relative levels of *tatn-1* expression were determined using the 2^−ΔΔCt^ approach [96], [97].

The oligos used to detect *pmp-3*, *cdc-42*, and *Y45F10D.4* have been previously described [98]. The expression of *sod-3* was detected using the oligos 5′-CCAACCAGCGCTGAAATTCAATGG-3′ and 5′-GGAACCGAAGTCGCGCTTAATAGT-3′ [99]. The expression of *tatn-1* was detected using 5′-CTTGATCAGAGAAGAATCAGTG-3′ and 5′-GAGTGTTGATTGAAGTTGCG-3′. These oligos were designed to cross intron-exon boundaries using the PerlPrimer program [100].

### Whole transcriptome RNA sequencing

N2 wild-type control and *tatn-1(qd182)* mutant worms were synchronized via the use of hypochlorite treatment and grown on HB101 spotted NGM plates at 25°C for 2 days. These conditions and time point correspond to the conditions used for the amino acid analysis separately performed using these strains. The worms were washed from the plates and were then washed twice with miliQ water. The worm pellet was then suspended in QIAzol lysis reagent (Qiagen, Valencia, CA) and frozen at −80°C. Total RNA was isolated using the Qiagen miRNeasy mini kit and the RNA yield was measured by spectrophotometry. Total RNA was sent to Expression Analysis (Durham, NC) for analysis including bioanalyzer electrophoresis to ensure RNA quality followed by library preparation using the Illumina TruSeq RNA sample prep kit. The resulting library was subjected to high-throughput 50 nucleotide paired end sequencing using an Illumina sequencer at a depth of 17 million reads per sample.

The resulting sequence data was clipped using internally developed software from Expression Analysis and matched to the *C. elegans* genome using RSEM [101]. The resulting transcript counts were then normalized using the upper quartile normalization approach [102]. Differentially expressed genes were then identified through the use of ANOVA testing and genes with a FDR score of 5% or lower were considered to be differentially expressed. This lead to the identification of 4622 genes as being differentially expressed (890 up-regulated and 3732 down-regulated) between *tatn-1(qd182)* and wild-type N2.

Over-represented gene classes were identified in the up-regulated and down-regulated genes through the use of DAVID [60]. Analysis of the transcriptome data for *daf-16*/FOXO and *crh-1*/CREB regulated genes was performed using the Gene Set Association Analysis for RNA-seq program (available at http://gsaa.unc.edu/login/index.html) [63]. GSAA calculates a differential expression score for each gene in the entire RNA-seq dataset, 20408 genes in all, and then uses a running weighted Kolmogorov-Smirnov test to examine association of an entire gene set with each phenotypic class. The strength of the association is measured by the association score (AS) where positive scores indicate association of the gene set with the phenotype, and statistical significance is measured by a false discovery rate (FDR) that is adjusted for multiple testing. The *daf-16*/FOXO regulated genes were from previously published microarray data from Murphy et. al., and the *crh-1*/CREB regulated genes were from recently published microarray data from Mair et. al. [53], [62].

### Generation of TATN-1:GFP transgenic animals

Clones for the *tatn-1* promoter and cDNA where purchased from Open Biosystems and verified by sequencing [103], [104]. A *tatn-1p:tatn-1 cDNA:GFP* transgene was generated using Gateway cloning and the vector pDEST-MB14 [104]. The resulting transgene or p*unc-119cbr*, which contains the *unc-119* gene from *Caenorhabditis briggsae*, was used to bombard HT1593 (*unc-119(ed3)*) as previously described [105], [106]. From bombardment we obtained *bafIs130* from p*unc-119cbr*, which carries the *unc-119* gene from *Caenorhabditis briggsae*, and *bafIs131* from the *tatn-1p:tatn-1 cDNA:GFP* transgene [106]. Both transgenes were outcrossed with N2 and then mated with *eak-4(mg348); tatn-1(baf1)* to test for rescue of the dauer formation phenotype. The *bafIs131* transgene was also crossed into a *daf-2(e1368)* mutant to generate *daf-2(e1368); bafIs131*.

### Anti-phospho AMPK western blotting

Worm embryos were isolated by sodium hypochlorite treatment, and eggs were transferred to NGA plates spotted with HB101 and incubated at 25°C for 24 hours. For treatment with AICAR, the plate was spotted 1 hour before adding the eggs with aliquots from a 250 mM AICAR stock that was dissolved in water. As a positive control, N2 wild-type worms were grown as above, washed off in S-basal, and then exposed to 10 mM sodium azide in S-basal. This dose of S-basal has been previously shown to result in a 50% decrease in ATP concentrations in treated worms [54]. The worms were washed from plates in water, pelleted by centrifugation, washed with water, and suspended in 1× LDS loading buffer (Invitrogen) before being heated to 70°C in a Bransonic sonicator waterbath (Branson) for 30–40 minutes [107]. After heating the samples were centrifuged and the supernatant retained for analysis. The protein levels were measured using the CB-X protein assay kit (G-Biosciences), and 30 µg of protein was run on a 10% Nupage SDS-PAGE gel (Invitrogen) and blotted to a nitrocellulose membrane. Phospho-AAK-2 was detected using a rabbit monoclonal antibody (Cell Signaling Technology #2535), and actin was detected using a rabbit anti-actin antibody (Cell Signaling Technology #4967) followed by detection by an anti-rabbit HRP secondary antibody and visualization using chemiluminescence (Bio-Rad). The resulting X-ray film was scanned and quantified using gel analysis tools in ImageJ [108].

## Supporting Information

Figure S1 The interaction between *tatn-1* and *eak-4* is modified by bacterial diet. Shown are the effects of HB101, HT115, OP50, or OP50-1 bacterial diets on dauer formation by *eak-4(mg408); tatn-1(baf1)* mutants. These results represent one of two trials, and the shadings represent the percentages of L2, Dauer, or L3/L4 or older animals within each population. ** p<0.001 for pairwise Fisher's exact contingency tests comparing OP50 versus HB101 or HT115 and OP50-1 versus HB101 or HT115.
(EPS)

Figure S2 The *tatn-1(qd182)* allele enhances dauer formation by *eak-4* mutants. (A) The point mutation in the *tatn-1(qd182)* allele produces a G to E change in a conserved residue of tyrosine aminotransferase as indicated by the arrow. This change is predicted to have an >89% chance of impairing protein function based on analysis using the coding SNP scoring tool available at the Panther database. (B) Enhanced dauer arrest by the *eak-4; tatn-1(qd182)* mutant compared to *eak-4* or *tatn-1(qd182)* alone, or the N2 wild type strain. ** p<0.001 for pairwise Fisher's exact test.
(EPS)

Figure S3 *tatn-1* acts independently of the TGF-β signaling pathway. Inhibiting the TGF-β-like signaling pathway involved in dauer formation with mutations affecting *daf-3*/SMAD or *daf-5*/Sno genes does not block dauer formation by the *eak-4; tatn-1* mutants.
(EPS)

Figure S4 The effects of the *tatn-1(qd182)* allele on dauer formation depend on *aak-2*/AMPK. (A) The effects of *tatn-1(qd182)* on dauer formation also require *aak-2*. ** p<0.001 by Fisher's exact test. (B) The *tatn-1(qd182)* allele is likely stronger than *tatn-1(baf1)* because the *tatn-1(qd182)* allele shows delayed development compared to *tatn-1(baf1)* and wild type N2 worms. The curves show the percentage of adults present at each timepoint after synchronization. (C) SDS selection, which kills animals which have not fully completed dauer development including synthesis of the dauer cuticle and cessation of pharyngeal pumping, reveals a stronger effect of *aak-*2/AMPK mutations on dauer formation by the *eak-4(mg348)*; *tatn-1(qd182)* mutants compared with scoring based on morphology.
(EPS)

Figure S5 *tatn-1* mutations do not impair energy production and increase the AMP/ATP ratio. L2 larval wild-type N2 and *eak-4(mg348); tatn-1(baf1)* worms were collected and nucleotides were extracted for measurement using HPLC with UV detection. Shown is the average AMP/ATP ratio for three separately grown worm preparations (N2 mean 0.036 and *eak-4; tatn-1* mean 0.029, p = 0.73 by t-test).
(EPS)

Figure S6 The effects of the *tatn-1(qd182)* allele on dauer formation depend on *daf-16*/FOXO. (A) Loss of *daf-16* in the *daf-16(mgDf47)* mutant partially inhibits dauer formation by *eak-4(mg348); tatn-1(qd182)* based on morphology, but SDS selection, which kills larvae which have not fully completed dauer development (B) reveals a stronger inhibitory effect of the *daf-16(mgDf47)* mutation on dauer formation.
(EPS)

Figure S7 Pie charts representing gene ontology categories of differentially expressed genes in the *tatn-1(qd182)* mutant compared to wild-type N2. Genes as being differently expressed where divided into up-regulated and down-regulated groups and then analyzed using the tools within the Panther database. Pie charts were generated for each group using the “biologic process”, “cellular component”, and “molecular function” gene ontology perspectives.
(EPS)

Figure S8 *daf-2*/IGFR signaling inhibits the expression of *tatn-1* mRNA. Measurement of *tatn-1* mRNA levels in N2 and *daf-2(e1368)* mutants reveals that the *daf-2* mutants show a 40–50% increase in *tatn-1* expression compared to N2. Shown are the results of two independent trials using RNA isolated from *daf-2* and N2 adult animals.
(EPS)

Figure S9 Tyrosine supplementation raises tyrosine levels but does not affect worm lifespan. (A) Growth of N2 worms on NGA supplemented with 1 mg./mL tyrosine leads to an increase in tyrosine levels as shown by liquid chromatography and tandem mass spectrometry. The untreated N2 average is 74.3 µmol per 100 worms and the tyrosine treated average is 163.6 µmol per 100 worms, which is a 2.2 fold increase. (B) Lifespan assays performed with N2 worms supplemented with tyrosine, glycine, or isoleucine show no effect of tyrosine supplementation on lifespan compared to untreated N2 animals (Untreated N2 mean survival 19.5 days, tyrosine treated 20.0 days, glycine treated 19.0 days, and isoleucine treated 21.5 days). The isoleucine treatment produces a small, but consistent effect on worm lifespan in two separate trials.
(EPS)

Table S1 Function and human homologs for genes studied. Table showing the function and human homolog identified using HomoloGene, or Wormbase if not hits were identified, for each of the genes studied.
(DOCX)

Table S2 Effects of *tatn-1* on dauer formation. Excel spreadsheet showing the genetic and dauer formation assay data used to create each panel.
(ZIP)

Table S3 Effects of *tatn-1* on longevity. Excel spreadsheet showing the lifetable analysis for lifespan experiments shown in Figure 2, 3, 5, and 6.
(ZIP)

Table S4 Genes differentially expressed between *tatn-1(qd182)* mutants and N2 worms. Excel spreadsheet showing genes identified as up-regulated or down-regulated at a 5% false discovery rate through RNA-seq experiments with three *tatn-1(qd182)* and three wild-type N2 RNA samples.
(ZIP)

Table S5 Gene classes identified as enriched via use of the DAVID program. The lists of up-regulated and down-regulated genes where searched for evidence of enriched functional or structure gene classes via use of the DAVID computer program. Shown is an Excel spreadsheet showing the DAVID output for each gene list.
(ZIP)

Table S6 List of oligos and enzymes used to genotype progeny of crosses. Excel spreadsheet containing the oligo sequences and when needed restriction enzymes used to genotype the progeny of crosses involving the listed mutations. For all experiments, multiple rounds of self-fertilization and PCR genotyping were used to identify progeny homozygous for all mutations.
(ZIP)

Table S7 Correlation between raters for a set of control images depicting worm larval stages. A group of raters independently viewed a series of 60 images and accompanying movies of larval worms of differing developmental stages in random order. Raters scored each animal as L2 or below, dauer, or L3 or above. Correlation between the raters was determined via the kappa statistic. Note that one rater had never previously worked with *C. elegans* and learned to perform scoring via a brief tutorial prior to scoring the image set.
(DOCX)
