# Supplementary material for: TATN-1 Mutations Reveal a Novel Role for Tyrosine as a Metabolic Signal That Influences Developmental Decisions and Longevity in Caenorhabditis elegans
Source: PLoS Genet. 2013 Dec 19;9(12):e1004020. doi: 10.1371/journal.pgen.1004020 (PMC3868569; doi:10.1371/journal.pgen.1004020)
Supplement: Table S1 — Function and human homologs for genes studied. Table showing the function and human homolog identified using HomoloGene, or Wormbase if not hits were identified, for each of the genes studied. (DOCX) [file pgen.1004020.s010.docx]

| ***C.elegans* Gene** | **Function** | **Human Ortholog (from HomoloGene)** |
| --- | --- | --- |
| *daf-2*  *age-1*  *pdk-1*  *daf-16*  *daf-7*  *daf-9*  *daf-11*  *tatn-1*  *eak-4*  *eak-3*  *sdf-9*  *hsd-1*  *eak-7*  *sgk-1*  *aak-2*  *daf-12*  *crh-1*  *pah-1*  *hpd-1*  *cat-2*  *tdc-1* | Insulin/Insulin-like growth factor-1 receptor  Phosphatidylinositol-4-phosphate 3-kinase  3-phosphoinositide-dependent protein kinase  Forkhead box transcription factor  TGF-β family member  Cytochrome P450  Guanylyl Cyclase  Tyrosine Aminotransferase  Unknown  Unknown  Tyrosine phosphatase-like protein  3β-hydroxysteroid dehydrogenase  TLD domain-containing protein  Serum/glucocorticoid regulated kinase  AMP kinase  Nuclear hormone receptor  CREB transcription factor  Phenylalanine-4-hydroxylase  4-hydroxyphenylpyruvate dioxygenase  Tyrosine hydroxylase  Tyrosine decarboxylase | IGF1R/INSR PIK3CA PDPK1 FOXO1GDF11 CYP2W1 GUCY2D TAT  None  RNF11  None  NSDHL TLDC1SGK1 PRKAA2  NR1H2 CREB1/CREM PAH HPDTHNone |
