# Supplementary material for: TATN-1 Mutations Reveal a Novel Role for Tyrosine as a Metabolic Signal That Influences Developmental Decisions and Longevity in Caenorhabditis elegans
Source: PLoS Genet. 2013 Dec 19;9(12):e1004020. doi: 10.1371/journal.pgen.1004020 (PMC3868569; doi:10.1371/journal.pgen.1004020)
Supplement: Table S7 — Correlation between raters for a set of control images depicting worm larval stages. A group of raters independently viewed a series of 60 images and accompanying movies of larval worms of differing developmental stages in random order. Raters scored each animal as L2 or below, dauer, or L3 or above. Correlation between the raters was determined via the kappa statistic. Note that one rater had never previously worked with C. elegans and learned to perform scoring via a brief tutorial prior to scoring the image set. (DOCX) [file pgen.1004020.s016.docx]

|  | Rater 1 | Rater 2 | Rater 3 | Rater 4 |
| --- | --- | --- | --- | --- |
| Rater 1 |  | 0.8997 | 0.7468 | 0.8739 |
| Rater 2 |  |  | 0.7964 | 0.8236 |
| Rater 3 |  |  |  | 0.7911 |
| Rater 4 |  |  |  |  |

Pairwise kappa statistic values between four raters were calculated using the FREQ procedure in SAS version 9.3. Each comparison had a kappa value designated as “substantial” to “almost perfect” agreement [92].
